# High-Pressure Vibrational and Structural Properties of Ni_3_V_2_O_8_ and Co_3_V_2_O_8_ up to 20 GPa

**DOI:** 10.1021/acs.jpcc.3c04019

**Published:** 2023-10-30

**Authors:** Josu Sánchez-Martín, Julio Pellicer-Porres, Akun Liang, Jordi Ibáñez, Robert Oliva, Catalin Popescu, Zhangzhen He, Plácida Rodríguez-Hernández, Alfonso Muñoz, Daniel Errandonea

**Affiliations:** † Departamento de Física Aplicada-ICMUV, MALTA-Consolider Team, Universidad de Valencia, Dr. Moliner 50, Burjassot, Valencia 46100, Spain; ‡ CSEC, The University of Edinburgh, UoE, School of Physics and Astronomy, Edinburgh EH9 3FD, United Kingdom; § MALTA-Consolider Team, Geosciences Barcelona (GEO3BCN), CSIC, Lluís Solé i Sabarís s/n, 08028 Barcelona, Spain; ∥ CELLS-ALBA Synchrotron Light Facility, MALTA-Consolider Team, Cerdanyola del Vallès, Barcelona 08290, Spain; ⊥ State Key Laboratory of Structural Chemistry, Fujian Institute of Research on the Structure of Matter, Chinese Academy of Sciences, Fuzhou, Fujian 350002, China; # Departamento de Física, MALTA-Consolider Team, Instituto de Materiales y Nanotecnología, Universidad de La Laguna, San Cristóbal de La Laguna, Tenerife E-38200, Spain

## Abstract

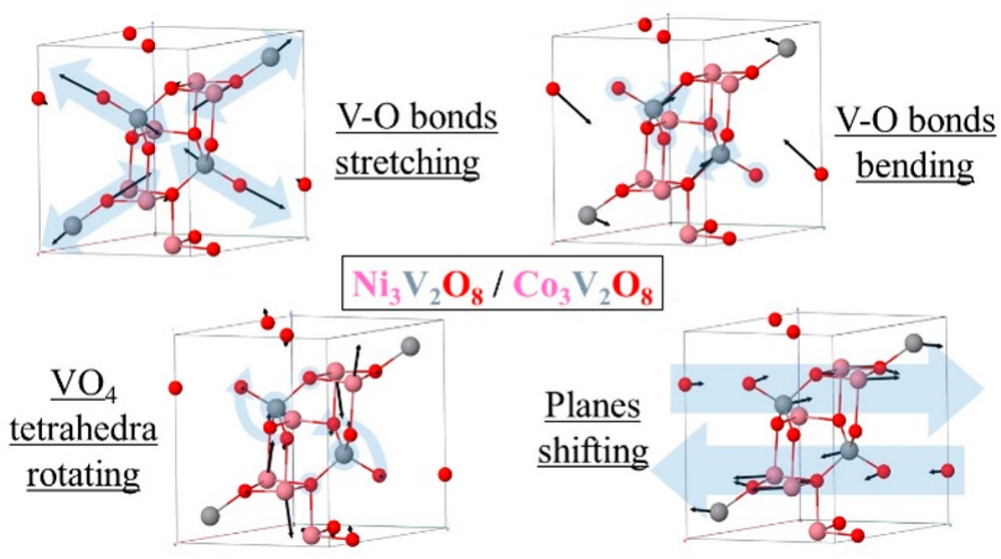

The vibrational and structural behaviors of Ni_3_V_2_O_8_ and Co_3_V_2_O_8_ orthovanadates have been studied up to around 20 GPa by means of X-ray diffraction, Raman spectra, and theoretical simulations. Both materials crystallize in an orthorhombic Kagomé staircase structure (space group: *Cmca*) at ambient conditions, and no phase transition was found in the whole pressure range. In order to identify the symmetry of the detected Raman-active modes under high pressure, single crystal samples of those materials were used in a polarized Raman and infrared setup. Moreover, high-pressure powder X-ray diffraction measurements were performed for Co_3_V_2_O_8_, and the results confirmed the structure stability also obtained by other diagnostic techniques. From this XRD analysis, the anisotropic compressibilities of all axes were calculated and the unit-cell volume vs pressure was fitted by a Birch–Murnaghan equation of state, obtaining a bulk modulus of 122 GPa.

## 1 Introduction

Metal orthovanadates following the formula M_3_V_2_O_8_ (M = Ni, Co, Zn, Mn, and Mg) have attracted considerable fundamental research attention for decades,^1−3^ due to their rich polymorphism^4−7^ and multiferroic properties.^8−10^ These qualities make them desirable materials for industrial applications. Regarding the samples studied in this work, Ni and Co orthovanadates are mainly used in nanostructured systems. Both have been investigated as catalysts in the water splitting process,^11,12^ as electrodes in portable power sources,^13,14^ in the potential improvement of electrochemical energy storage,^15,16^ in nitrogen fixation,^17^ and even in glucose detection.^18^

**Figure 1:**
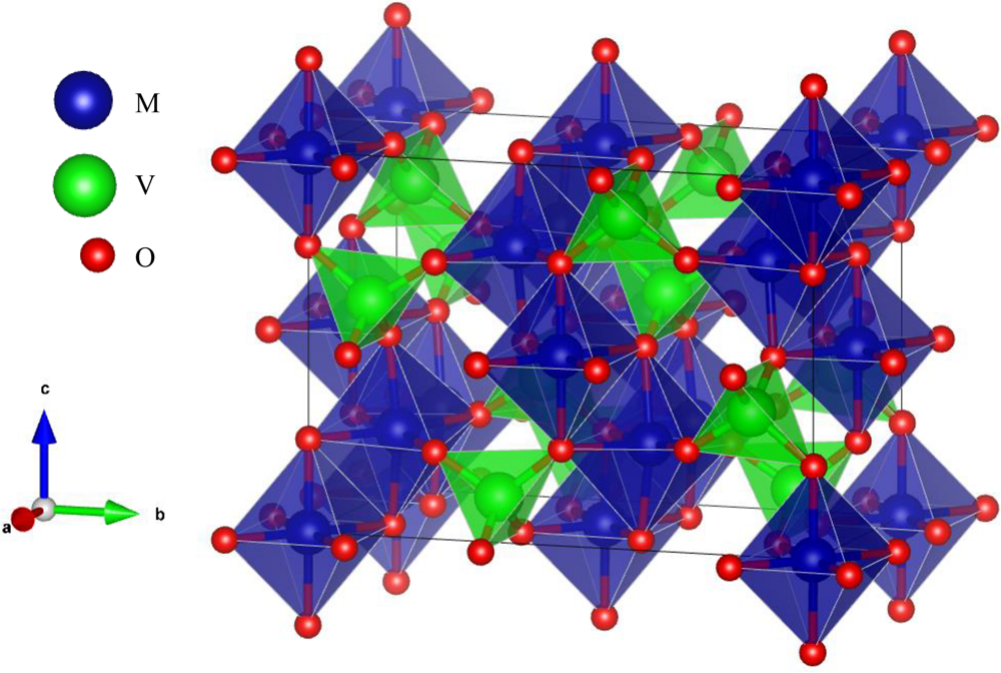
Crystal structure of the M_3_V_2_O_8_ orthovanadate Kagomé-staircase family. Atoms and unit-cell axis are labeled on the left. MO_6_ octahedra are shown in blue, and VO_4_ tetrahedra are shown in green.

The so-called Kagomé-staircase orthorhombic structure of Ni_3_V_2_O_8_ and Co_3_V_2_O_8_ (space group: *Cmca*, No. 64) is formed by corrugated layers in the [010] direction of edge-sharing MO_6_ octahedra interconnected with VO_4_ tetrahedra (see Figure 1). Both compounds have four formulas per unit cell (*Z* = 4). The lattice parameters for Ni_3_V_2_O_8_ are *a* = 5.936(4) Å, *b* = 11.420(6) Å, and *c* = 8.240(5) Å and for Co_3_V_2_O_8_ are *a* = 6.030(4) Å, *b* = 11.486(2) Å, and *c* = 8.312(5) Å.^1^ It is worth mentioning that a similar polyhedral coordination is also found in metavanadates (MV_2_O_6_)^19^ and pyrovanadates (M_2_V_2_O_7_).^20^

In recent years, the high pressure (HP) community has also put the focus on this family of compounds, which have demonstrated a variety of remarkable physical behaviors under pressure. X-ray diffraction (XRD) and Raman methods were used to study Mn_3_V_2_O_8_ in its orthorhombic low-temperature structure; an irreversible phase transition at 10 GPa was discovered, but the new phase has not been identified yet.^21^ In contrast, it has been demonstrated that Zn, Ni, and Mg orthovanadates are stable up to 15,^22^ 23,^23^ and 25.7 GPa,^24^ respectively. With different structures but the same stoichiometry, Ca and Sr orthovanadates were found to undergo different phase transitions at 9.7(1) GPa^25^ and 13.8 GPa,^26^ respectively. Alternatively, it was reported that triclinic Cu_3_V_2_O_8_ decomposes into CuO and V_2_O_5_ at 1.35 GPa.^27^ Note that the structural properties of Ni_3_V_2_O_8_ were recently investigated by HP powder XRD by some of the authors of the present study.^24^ To compare with Co_3_V_2_O_8_, the data from that work are included in the current investigation.

We continue the research in orthovanadates within this paper by reporting for the first time the changes in the vibrational modes of Ni_3_V_2_O_8_ and Co_3_V_2_O_8_ under HP from both experiments and density functional theory (DFT) calculations. As a previous step, polarized Raman and infrared (IR) measurements are used to properly identify the symmetry of the active modes and match them with the simulation results under ambient conditions. We also present the first HP XRD analyses of Co_3_V_2_O_8_. As it is situated in the periodic table between Mn (which undergoes a phase transition at 10 GPa^21^) and Ni (which remains stable up to 23 GPa^23^), it is of great interest to find out what structural changes occur under pressure. The experimental data are also supported by the corresponding DFT calculations. We determined the bulk modulus and anisotropic compressibility of this compound from the structural information we have collected. Finally, we make use of all the results obtained to compare the pressure behavior of both orthovanadates.

## 2 Methods

### 2.1 Sample Synthesis

Powder samples of Ni_3_V_2_O_8_/Co_3_V_2_O_8_ were synthesized by means of a solid-state reaction starting with NiO/CoO (99.995% purity) and V_2_O_5_ (99.9% purity). The precursors were obtained from Alfa Aesar. An Al_2_O_3_ crucible was used to heat the mixed reagents in air at 800 °C for 16 h. The product was then ground and pressed into a pellet, which was sintered at 900 °C for an additional 16 h.

For the single crystal preparation, Ni_3_V_2_O_8_/Co_3_V_2_O_8_ powders were prepared at 900 °C for 40 h by a standard/high-temperature solid-state reaction method using NiC_2_O_4_·2H_2_O/CoC_2_O_4_·2H_2_O and V_2_O_5_ as the reagents with a molar ratio of 3:1. The crystal growth was performed in an electric furnace, where Ni_3_V_2_O_8_/Co_3_V_2_O_8_ powder samples and flux V_2_O_5_ and SrCO_3_ (also BaCO_3_ for Co_3_V_2_O_8_) were melted homogeneously in an alumina crucible at 1000 °C and kept at 1000 °C for 10 h, cooled slowly to 800 °C/700 °C at a rate of 0.5 °C/h (making constant temperature stops several times in between), and finally cooled to room temperature at a rate of approximately 100 °C/h. The final Ni yellow crystals (∼3 × 3 × 0.5 mm^3^)/Co dark blue crystals (∼4 × 4 × 1 mm^3^) were obtained by mechanical separation from the crucible. A detailed growth procedure is described in ref (28) for Ni_3_V_2_O_8_ and in ref (29) for Co_3_V_2_O_8_.

### 2.2 Experimental Details

The orientation of the single crystals was carried out by using a Bruker D8 Venture diffractometer. IR spectra at ambient conditions were collected with an FTIR Bruker IFS125 HR spectrometer using a Globar light source, KBr beam splitter, and MCT detector (cut at 600 cm^–1^). Raman spectra were acquired in the backscattering geometry using a 632.8 nm He–Ne laser, a Jobin Yvon spectrometer combined with a thermoelectric-cooled multichannel charge-coupled device (CCD) detector with a spectral resolution of 2 cm^–1^, and a Semrock low-pass RazorEdge filter. A low laser power of approximately 2 mW before the diamond anvil cell (DAC) was necessary to avoid overheating the sample and wavenumber shifting. Polarizer filters were added to the Raman setup for the single crystal measurements. HP Raman measurements were performed using a DAC and a 16:3:1 methanol–ethanol–water mixture as the pressure-transmitting medium (PTM).^30^ The peak profile fit was achieved using a Pseudo-Voight peak profile in MATLAB software.^31^ The pressure gauge was determined using ruby luminescence.^32^

HP powder XRD measurements on Co_3_V_2_O_8_ were performed at the MSPD beamline of the ALBA synchrotron^33^ using a monochromatic beam with a wavelength of 0.4246 Å. The beam was focused down to a spot with a full width at half-maximum (fwhm) of 20 μm × 20 μm. A Rayonix CCD detector was used to collect XRD patterns with a sample-to-detector distance of 340 mm. This sample–detector distance was required to achieve a correct angular resolution, which limited our 2θ range to around 13°. The pressure was determined using the XRD reflections and the equation of state (EOS) of Cu^34^ with a precision of ±0.1 GPa. The PTM used for these experiments was a 4:1 methanol–ethanol (ME) mixture. The measurements thus obtained were transformed into one-dimensional patterns using the DIOPTAS suite,^35^ and Le Bail fittings were achieved with PowderCell.^36^

### 2.3 Ab Initio Density-Functional Theory Calculations

Ab initio calculations were performed within the framework of density functional theory (DFT)^37^ with the Vienna ab initio Simulation Package (VASP).^38,39^ The projector augmented-wave (PAW) method^40,41^ was employed. To ensure accurate converged results, the plane-wave kinetic cutoff was extended up to 650 and 540 eV for Ni_3_V_2_O_8_ and Co_3_V_2_O_8_, respectively. The integrations over the Brillouin zone (BZ) were carried out with a k-special point sampling grid of 6 × 6 × 4. After testing different functionals to decide which was the most accurate for each compound, the exchange-correlation energy was described by means of the generalized gradient approximation (GGA) with the Armiento and Mattsson (AM05) prescription^42,43^ for Ni_3_V_2_O_8_ and, in the case of Co_3_V_2_O_8_, the Perdew–Burke–Ernzerhof (PBE) functional for solids.^44,45^ To properly treat the strongly correlated states, the DFT + U method of Duradev et al.^46^ was employed. This method utilizes a single parameter, *U*_eff_ = *U* – *J*, where *U* and *J* are the effective on-site Coulomb and exchange parameters, respectively. The values used for *U*_eff_(47) were 6.2 eV for Ni, 3.25 eV for V, and 3.32 eV for Co. In both compounds, the ferromagnetic configuration was found to be lower in energy.

The unit cell parameters and atomic positions were fully optimized to obtain, at selected volumes, the relaxed structure. For the optimization, the criteria used were as follows: the forces on the atoms were less than 3 meV/Å, and the deviations of the stress tensors from a diagonal hydrostatic form were lower than 0.1 GPa. Our ab initio calculations provide a data set of volumes, energies, and pressures (from the stress tensor) that are fitted with a Birch–Murnaghan equation of state^48^ to obtain the theoretical equilibrium volume, the bulk modulus, and the pressure derivatives.

Lattice-dynamic calculations of the phonon modes were carried out at the zone center (Γ point) of the BZ with the direct force-constant approach provided by Phonopy.^49^ These calculations provide the frequency of the normal modes, their symmetry, and their polarization vectors. This allows the identification of the irreducible representations and character of the phonon modes at the Γ-point. To include the polarization induced by atomic displacements and the generated macroscopic electric field producing the LO/TO splitting, the nonanalytical term corrections were added using a 2 × 2 × 2 supercell, with the Born effective charges and the dielectric tensor as described in the Phonopy package.^49^

## 3 Results and Discussion

### 3.1 Vibrational Properties of Ni_3_V_2_O_8_ and Co_3_V_2_O_8_

Both Ni and Co orthovanadates present the same crystalline structure,^1^ whose symmetry is described by the *Cmca* space group. There are two molecules in the primitive unit cell, giving rise to seventy-eight vibrational modes. Point group *mmm* classifies the symmetry at the zone center as follows^50^:
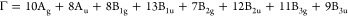


Even (gerade) modes (A_g_, B_1g_, B_2g_, and B_3g_) are Raman active. Ni/Co atoms located at inversion centers (those at the 4a Wyckoff position) remain at rest. One of each of the B_1u_, B_2u_, and B_3u_ modes corresponds to acoustic modes. The rest are IR active modes with the exception of A_u_ modes, which are silent. All these modes have been individually labeled in Tables 1 (IR active) and 4 (Raman active). The calculated atomic motions of all vibrational modes are represented in Tables S1, S2, and S3.

**Table 1:** Ab Initio Calculated IR Modes under Ambient Conditions^a^

	Ni_3_V_2_O_8_			Co_3_V_2_O_8_		
DFT mode	ω_0_ (TO)	∂ω/∂*P* (TO)	ω_0_ (LO)	ω_0_ (TO)	∂ω/∂*P* (TO)	ω_0_ (LO)
B_1u_^1^	144	0.6(1)	145	124	–0.04(1)	125
B_3u_^2^	150	0.9(1)	151	144	0.9(1)	144
B_2u_^2^	174	1.0(1)	174	155	0.4(1)	154
B_1u_^3^	186	1.1(1)	186	183	–0.3(1)	184
B_3u_^3^	197	1.3(1)	198	184	0.9(1)	185
B_2u_^3^	198	0.6(1)	199	182	–0.5(1)	182
B_1u_^4^	216	1.1(1)	218	200	0.8(1)	204
B_2u_^4^	221	1.4(1)	221	209	–0.8(1)	210
B_3u_^4^	245	6.8(1)	261	245	1.8(1)	258
B_1u_^5^	255	3.0(1)	264	247	–0.2(1)	249
B_2u_^5^	290	3.5(1)	308	278	2.0(1)	288
B_1u_^6^	301	2.2(1)	303	285	2.6(1)	288
B_3u_^5^	308	3.8(1)	308	293	4.0(1)	293
B_1u_^7^	312	2.6(1)	322	305	4.8(1)	313
B_2u_^6^	316	1.6(1)	317	301	1.6(1)	303
B_1u_^8^	322	4.8(1)	324	332	4.2(1)	330
B_2u_^7^	323	4.6(1)	339	320	4.1(1)	339
B_3u_^6^	332	4.1(1)	343	304	1.8(1)	307
B_1u_^9^	369	5.1(1)	376	342	4.3(1)	354
B_3u_^7^	372	4.1(1)	387	353	5.6(1)	365
B_2u_^8^	404	2.9(1)	406	387	2.6(1)	390
B_2u_^8^	414	4.5(1)	425	396	3.6(1)	410
B_2u_^9^	442	3.5(1)	446	421	3.7(1)	422
B_1u_^10^	449	3.5(1)	449	427	3.6(1)	427
B_1u_^11^	653	6.5(1)	710	636	6.4(1)	691
B_3u_^10^	664	6.6(1)	740	642	6.5(1)	716
B_1u_^9^	793	2.6(1)	872	806	4.4(1)	860
B_2u_^12^	796	6.5(1)	806	770	2.5(1)	779
B_2u_^11^	814	4.4(1)	855	790	5.9(1)	823
B_1u_^13^	821	2.8(1)	895	820	2.1(1)	874
B_2u_^12^	883	3.8(1)	917	864	2.7(1)	893

a Wavenumber (ω_0_) is expressed in cm^–1^ and pressure (*P*), in GPa. The DFT-calculated ω_0_ has a related uncertainty of ±5%.

**Figure 2:**
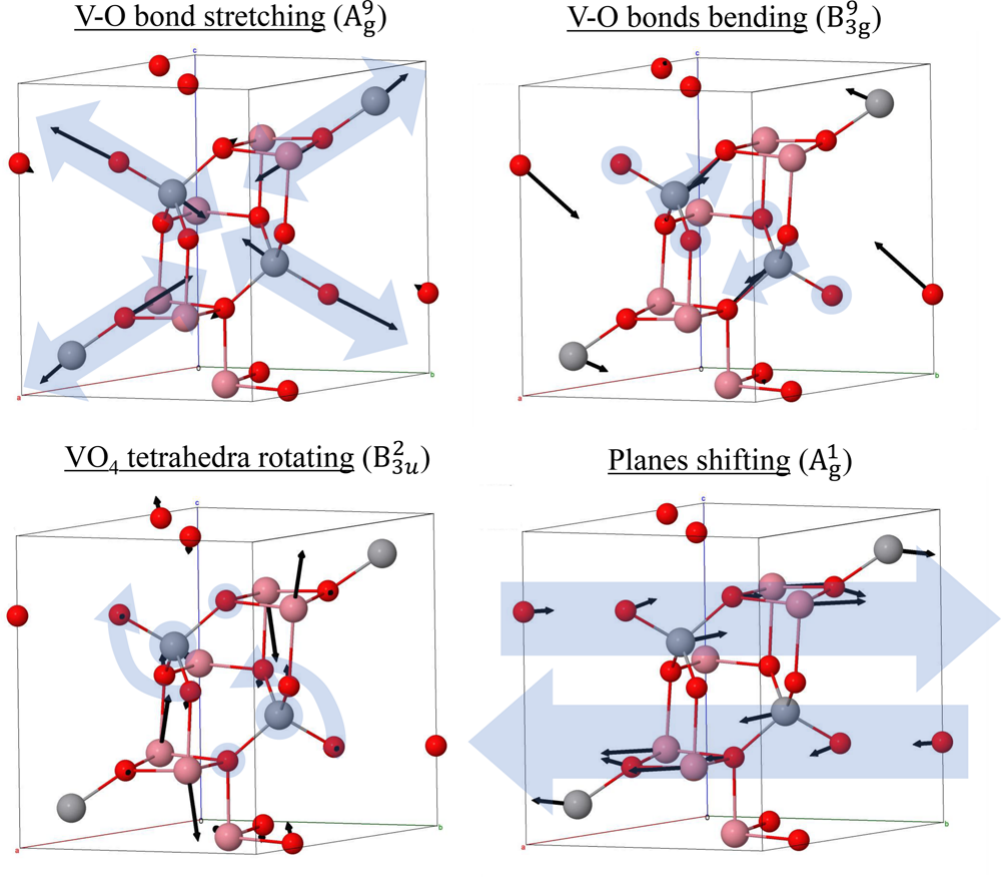
Representative vibrational modes of M_3_V_2_O_8_ (M = Ni, Co) using the primitive unit cell. M is in pink, V in gray, and O in red. Blue arrows represent the key motion, while blue dots represent key atoms in still positions.

The modes with the largest wavenumbers are related to the internal modes of the VO_4_ tetrahedra. Taking a closer look into some of the representative modes (referring to the wavenumbers of Co_3_V_2_O_8_), it can be seen that A_g_^9^ (Figure 2) and B_1u_^12^ are V–O bond stretching modes. Both have very similar calculated frequencies (789 and 770 cm^–1^, respectively), because their vibration pattern is very similar, but the inversion center makes the equivalent V and O movement through this point in phase or in phase opposition. Other examples of phonons related to the internal movement of the tetrahedra are the B_3g_^9^, Figure 2, and B_2u_^10^ modes (670 and 642 cm^–1^, respectively). Their vibration pattern includes bending of V–O bonds. From 640 to 440 cm^–1^, there is a frequency gap that divides internal from external modes. The more energetic mode with a relevant Co amplitude is the A_u_^7^ mode at 460 cm^–1^. The amplitude is, however, not large enough to be appreciated in Table S3. It is remarkable that internal modes have similar frequencies in Co and Ni compounds (a difference of less than 4%). In external modes where the M amplitude is relevant, the wavenumber differences are more pronounced. The mode with the largest Co amplitude is the B_1u_^2^ mode. The wavenumbers in Co and Ni compounds differ by 16% (124 and 144 cm^–1^, respectively). The mode B_3u_^2^ (144 cm^–1^ in the Co compound) constitutes an example of rotation of the VO_4_ tetrahedron, in this case having a V–O bond as an axis, Figure 2. The mode also involves a significant shift of the Co atoms. Finally, the low wavenumber mode A_g_^1^ (111 cm^–1^, Figure 2) represents a mode where atoms in a plane roughly defined by *z* ≅ 0.25 vibrate in phase opposition respect to atoms in a *z* ≅ 0.75 plane, while atoms near *z* = 0 and 0.5 remain static. The large mass involved implies low frequency mode. Other similar modes are B_1u_^2^ (124 cm^–1^) and B_1g_^1^ (121 cm^–1^).

### 3.2 Ambient Conditions for Infrared Spectroscopy (Ni_3_V_2_O_8_ and Co_3_V_2_O_8_)

IR modes were identified using polarization and considering that B_1u_, B_2u_, and B_3u_ modes transform as *z*, *y*, and *x*, respectively. The ab initio calculated IR active modes, including the transversal optic (TO) and longitudinal optic (LO) splittings and the corresponding pressure coefficients, are reported in Table 1. Furthermore, in Table 2, using the theoretical IR phonon wavenumbers and the simulated static dielectric constants (ε_0_), the infinite dielectric constants (ε_∞_) were calculated using the Lyddane-Sachs Teller relation.^51^

**Table 2:** Ab Initio Calculated Diagonal Components of the Static and Infinite Dielectric Constants of Ni_3_V_2_O_8_ and Co_3_V_2_O_8_ in Ambient Conditions

	ε_0_^*xx*^	ε_0_^*yy*^	ε_0_^*zz*^	ε_0_^*xx*^	ε_0_^*yy*^	ε_0_^*zz*^
Ni_3_V_2_O_8_	5.1(3)	5.3(3)	5.3(3)	3.0(2)	2.8(1)	3.0(2)
Co_3_V_2_O_8_	6.0(3)	6.2(3)	6.0(3)	4.1(2)	3.5(2)	3.6(2)

**Figure 3:**
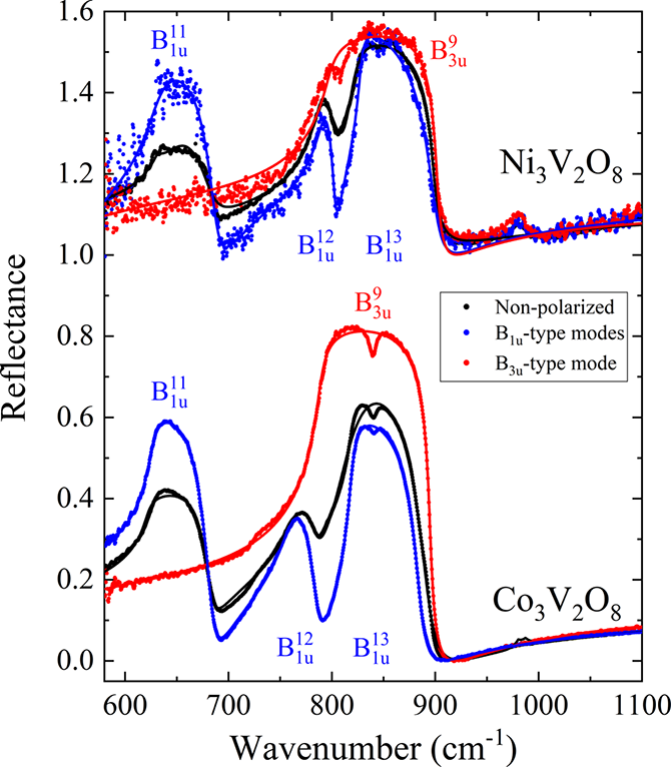
Experimental infrared reflectivity (dots) using 3 different incident polarizations. Solid lines represent the best fit^54^ for each set of data.

The growth conditions of the samples favored the formation of single crystals with the largest surface perpendicular to the *y*-axis. The measurements were taken on the [010] surface. The spectral region for the present IR measurements covered from 600 to 4500 cm^–1^. Therefore, only the three highest frequency B_1u_ modes and the last B_3u_ for both Ni_3_V_2_O_8_ and Co_3_V_2_O_8_ single crystals were accessible. These modes were selected using polarizers, as represented in Figure 3. The dielectric constant was modeled using the following relation:
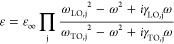
1where ω_TO_, ω_LO_, γ_TO_, and γ_LO_ are the frequencies and damping factors of the transverse and longitudinal optic modes, respectively.^52^ Using eq 1, the reflectivity of the material, , can be obtained, and then, the total reflectance of the sample can be calculated, considering that the body with parallel surfaces undergoes consecutive internal reflections, as follows:
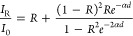
2where α is the absorption coefficient. Equation 2 was used to fit the experimental data in Figure 3. In the spectral region where the sample is transparent, this expression simplifies to
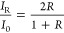
3

The criteria chosen for data normalization are based on the reflectance (3) at 4500 cm^–1^, where the sample is transparent and the reflectance is estimated using the calculated static dielectric constant from Table 2.

The experimentally determined IR modes are gathered along with the calculated modes in Table 3. It is noticeable that both experimental and theoretical values are in good agreement, including the TO-LO splitting values.

**Table 3:** Theoretical and Experimental Zone-Center IR Modes for Ni_3_V_2_O_8_ and Co_3_V_2_O_8_^a^

	DFT		experimental			
mode	ω_0_ (TO)	ω_0_ (LO)	ω_0_ (TO)	γ_0_ (TO)	ω_0_ (LO)	γ_0_ (LO)
Ni_3_V_2_O_8_						
B_1u_^11^	653	710	630(1)	24(4)	688(1)	16(3)
B_3u_^9^	793	872	797(1)	19(3)	902(1)	7(3)
B_1u_^12^	796	806	791(1)	14(6)	803(1)	8(4)
B_1u_^13^	821	895	828(1)	6(3)	901(1)	17(4)
Co_3_V_2_O_8_						
B_1u_^11^	636	691	626(1)	11(3)	684(1)	16(3)
B_3u_^9^	806	860	789(1)	10(3)	895(1)	6(4)
B_1u_^12^	770	779	759(1)	25(5)	787(1)	17(5)
B_1u_^13^	820	874	819(1)	8(4)	885(1)	12(5)

a γ_0_ is the damping factor of the fitting in cm^–1^, and ω_0_ is expressed in cm^–1^. The DFT-calculated ω_0_ has a related uncertainty of ±5%.

### 3.3 Ambient Conditions for Polarized Raman (Ni_3_V_2_O_8_ and Co_3_V_2_O_8_)

First, the Raman tensors of the allowed modes are^50^
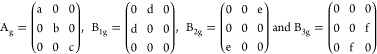


**Figure 4:**
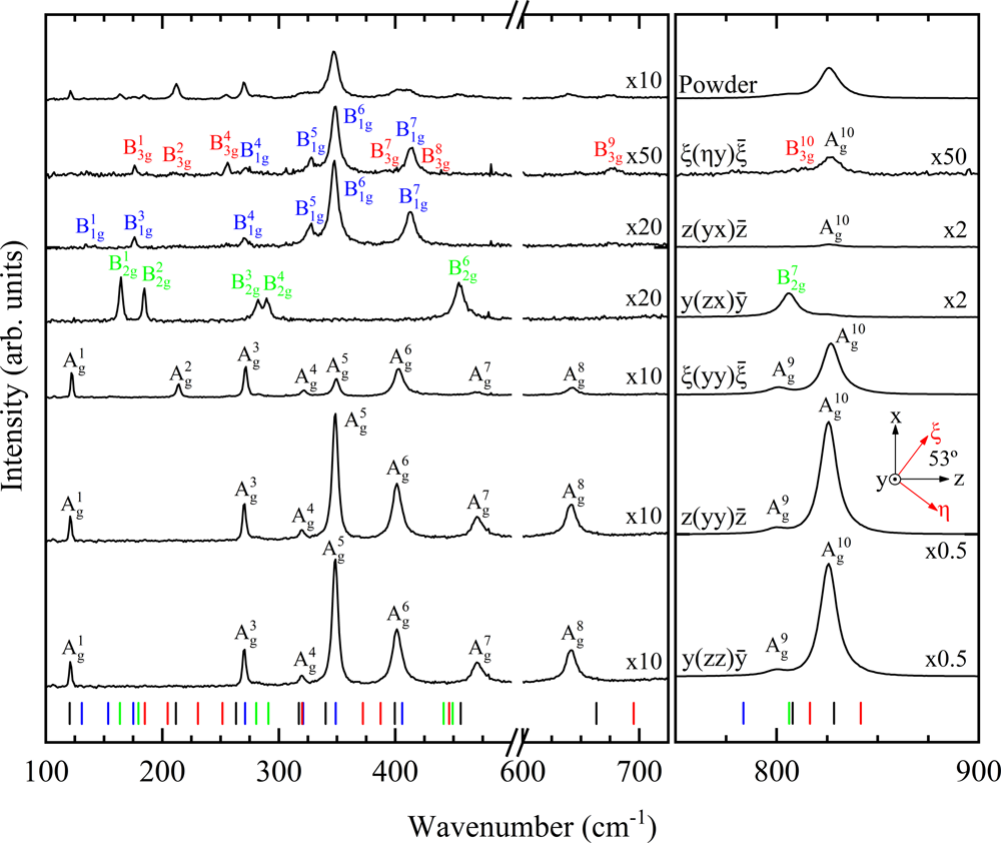
Symmetry assignment of the Raman modes of Ni_3_V_2_O_8_ at ambient conditions. The vertical ticks represent the DFT calculation results, matching in color with the corresponding symmetry. The incidence direction ξ of the tilted sample is sketched. If any magnification or reduction factor is applied to the data region, it is labeled next to it.

The resulting selection rules provide the advantage of being able to measure the modes separately, always in the backscattering configuration, employing the previously oriented single crystals. Additionally, it must be noted that A_g_ and B_1g_/B_2g_/B_3g_ modes are allowed when the backscattered signal from the single crystal sample is polarized parallel or perpendicular to the polarization of the incident laser, respectively. On the other hand, B_1g_/B_2g_/B_3g_ modes can only be measured if the surface of incidence is oriented in the crystallographic *c*/*b*/*a*-axis (hereon referred to as *z*/*y*/*x*). Depending on the specimen, other smaller surfaces different from [010] were also available. In the case of Ni_3_V_2_O_8_, a single crystal with a small [001] face was measured. In addition, a third perpendicular surface could be measured, starting from the [010] plane and tilting the DAC 53° with respect to the *z*-axis (from now on called the ξ orientation; see Figure 4), which yielded a spectrum containing B_1g_ and B_3g_ modes. In the case of Co_3_V_2_O_8_, only the [010] and [001] surfaces were available. The Raman characterization of both compounds at room temperature was completed with a powder spectrum.

**Figure 5:**
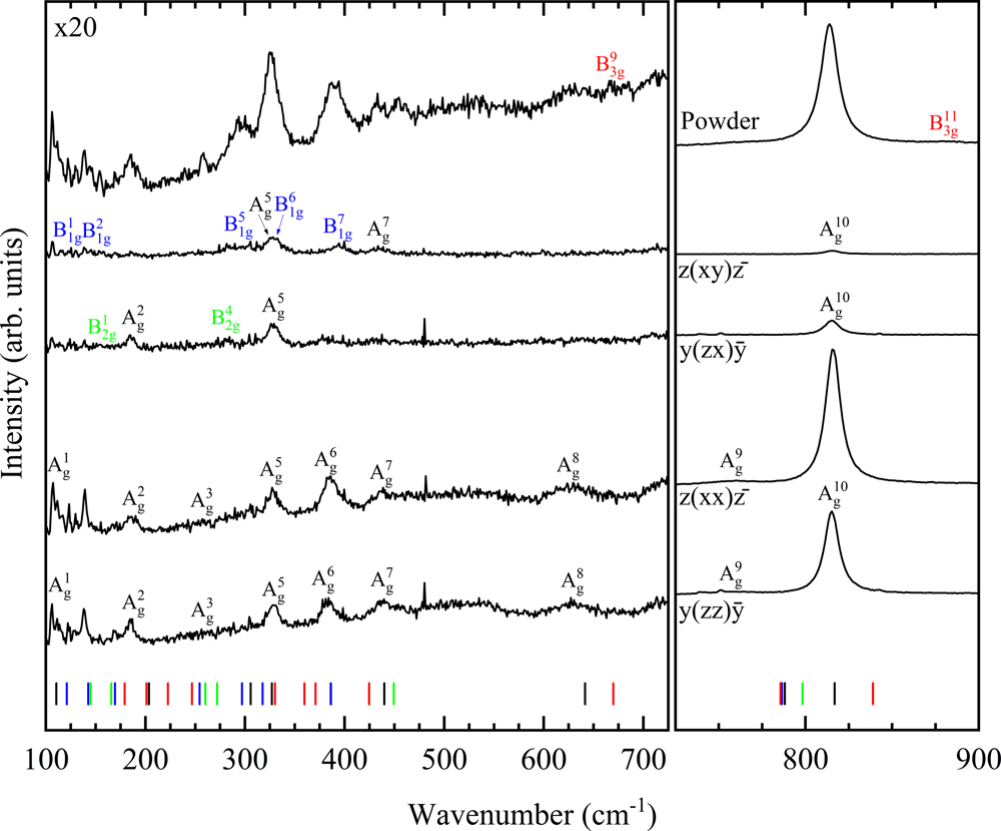
Symmetry assignment of the Raman modes of Co_3_V_2_O_8_ at ambient conditions. The vertical ticks represent the DFT calculation results, matching in color with the corresponding symmetry. The magnification of the low wavenumber region is 20×.

The complete symmetry phonon assignment for Ni_3_V_2_O_8_ is shown in Figure 4. All 10 A_g_ modes were found using coincident polarization in all 3 orientations of the crystal (except for A_g_^2^, which was found only in the ξ orientation). Using crossed polarization, the B_1g_ modes were identified when inciding along the *z*-axis (6 out of 8), B_2g_ modes, with *y*-incidence (6 out of 7), and a mixture of B_1g_ and B_3g_, in the ξ-axis (7 out of the 11 total B_3g_). There was no polarization leakage in any of the spectra, except for the most intense mode, A_g_^10^, which was also measured in crossed polarization at orientations *z* and ξ. Thus, a total of 29 modes of the 36 Raman-active modes are reported. Kesari et al. found 30 modes experimentally and performed DFT calculations.^53^ Overall, this work is in good agreement with this study, except that the symmetry of some nearby modes are not assigned in the same way (see B_1g_^3^-B_2g_^2^-B_3g_^1^, A_g_^4^-B_1g_^4^-B_2g_^3^, A_g_^5^-B_1g_^5^, B_1g_^6^-B_3g_^6^, A_g_^7^-B_3g_^8^, A_g_^9^-B_g_^6^, and A_g_^10^-B_3g_^11^ in Table 4), and not all the modes detected are the same. Combining both works, there are only 3 modes not detected experimentally. The analogue study for Co_3_V_2_O_8_ can be seen in Figure 5, which was less successful in comparison with the Ni compound due to its higher absorption of the excitation laser, giving rise to a sizably lower Raman signal. For this crystal, the parallel polarization measurements showed 9 of the total 10 A_g_ modes. Using crossed polarization, 5 B_1g_ modes were detected with *z* incidence and 2 B_2g_ modes, with *y* incidence. In all cases, small leaked contributions to A_g_ modes were found. Finally, all peaks from the polarized measurements were compared with those obtained from the powder sample, which allowed us to detect 2 extra modes belonging to the B_3g_ symmetry. The total amount of detected zone-center modes for the Co vanadate is 18 modes of the 36 available. Seo et al. were able to measure 8 modes of this compound,^54^ obtaining similar frequency values compared with this work. All of these mode identifications are supported by ab initio computations, showing satisfactory experiment–simulation agreement. The ambient pressure wavelength values of the vibrational modes for Ni_3_V_2_O_8_ and Co_3_V_2_O_8_, along with the calculated and literature values, are shown in Table 4.

**Table 4:** Raman Modes, Wavenumbers, and Pressure Coefficients Corresponding to the Zone-Center Active Raman Modes under Ambient Conditions for Ni_3_V_2_O_8_ and Co_3_V_2_O_8_^a^

	Ni_3_V_2_O_8_					Co_3_V_2_O_8_				
	this work		DFT		Kesari et al.^53^	this work		DFT		Seo et al.^54^
mode	ω_0_	∂ω/∂*P*	ω_0_	∂ω/∂*P*	ω_0_	ω_0_	∂ω/∂*P*	ω_0_	∂ω/∂*P*	ω_0_
A_1g_^g^	121(2)	0.1(5)	121	–0.1(1)	123	111(4)	0.6(1)	111	–0.2(1)	
B_1g_^1^	133(2)	0.8(3)	131	0.4(1)	135	123(5)	0.3(3)	121	0.4(1)	
B_1g_^2^			154	1.4(2)	157	138(4)	0.7(6)	143	1.4(1)	136
B_2g_^1^	164(3)	1.3(2)	164	1.3(1)	166	145(4)	1.8(4)	145	2.2(1)	
B_1g_^3^	171(6)	1.7(8)	175	0.7(2)	177 (B_3g_)			169	0.4(1)	
B_3g_^1^	176(3)	0.7(3)	185	0.9(1)	168 (B_2g_)			179	1.2(1)	
B_2g_^2^	184(2)	1.1(3)	180	1.4(1)	186 (B_1g_)			166	0.7(1)	
B_3g_^2^	209(3)	0.3(6)	205	0.3(2)	210			201	0.0(1)	
A_g_^2^	212(2)	2.5(5)	212	3.2(1)	213	185(3)	4.4(2)	204	2.6(1)	179
B_3g_^3^			231	1.0(1)	230			223	0.8(1)	
B_3g_^4^	255(2)	1.7(3)	252	2.0(1)	256			247	2.4(1)	
A_g_^3^	270(2)	0.9(6)	263	0.8(1)	271	258(4)	0.9(2)	260	0.4(1)	
B_1g_^4^	273(2)	3.9(3)	271	3.8(1)	328 (B_2g_)			254	3.7(1)	
B_2g_^3^	282(3)		281	4.0(1)	283 (A_g_)			260	4.3(1)	
B_2g_^4^	288(6)	4.2(2)	291	4.3(1)	291 (B_1g_)	282(5)		272	3.8(1)	
A_g_^4^	319(2)	5.6(2)	317	6.7(1)	320			306	2.3(1)	
B_3g_^5^			320	6.5(1)				330	8.0(1)	
B_1g_^5^	325(3)	3.8(3)	321	3.9(1)	351 (A_g_)	295(6)	5.8(2)	297	4.1(1)	
A_g_^5^	347(5)	2.8(7)	340	2.9(1)	348 (B_1g_)	337(3)	4.8(1)	327	3.2(1)	320
B_1g_^6^	351(2)	4.2(4)	349	5.0(1)	(B_3g_)	326(5)	4.7(1)	318	4.8(1)	
B_3g_^6^			372	2.3(1)	378 (B_1g_)			360	2.7(1)	
B_3g_^7^	392(7)		387	2.9(1)	390			371	2.8(1)	
A_g_^6^	400(6)	2.2(3)	400	1.9(1)	401	385(4)	1.5(1)	386	1.7(1)	384
B_1g_^7^	413(4)	3.7(2)	406	4.9(1)	413	394(5)	4.1(1)	386	4.3(1)	
B_2g_^5^			442	3.1(1)	455			425	3.1(1)	
B_3g_^8^	423(7)		446	2.7(1)	(A_g_)			425	2.3(1)	
B_2g_^6^	450(6)	6.4(2)	449	4.8(1)				449	4.5(1)	
A_g_^7^	458(4)	2.7(3)	456	2.9(1)	469 (B_3g_)	454(4)	2.7(1)	440	2.3(1)	450
A_g_^8^	640(6)	5.6(2)	663	6.5(1)	641	629(5)		642	6.5(1)	619
B_3g_^9^	675(4)	6.1(4)	695	5.8(1)	675	666(7)	4.3(7)	670	5.7(1)	666
B_1g_^8^			784	3.8(1)				786	2.2(1)	
B_2g_^7^	806(4)		806	3.8(1)	805 (A_g_)			798	2.7(1)	
A_g_^9^	805(3)	3.9(1)	808	5.6(1)	799 (B_2g_)	768(6)		788	7.6(1)	
B_3g_^10^	808(5)		817	5.6(1)	806			786	5.4(1)	
A_g_^10^	826(2)	2.6(1)	828	3.3(1)	825 (B_3g_)	814(2)	3.0(1)	817	2.2(1)	811
B_3g_^11^			842	3.5(1)	(A_g_)	880(7)		839	2.1(1)	

a ω_0_ is expressed in cm^–1^, and *P*, in GPa. The DFT-calculated ω_0_ has a related uncertainty of ±5%. Discrepancies in symmetry assignation with Kesari et al.^53^ are included in its column.

### 3.4 High-Pressure Raman Spectroscopy (Ni_3_V_2_O_8_ and Co_3_V_2_O_8_)

**Figure 6:**
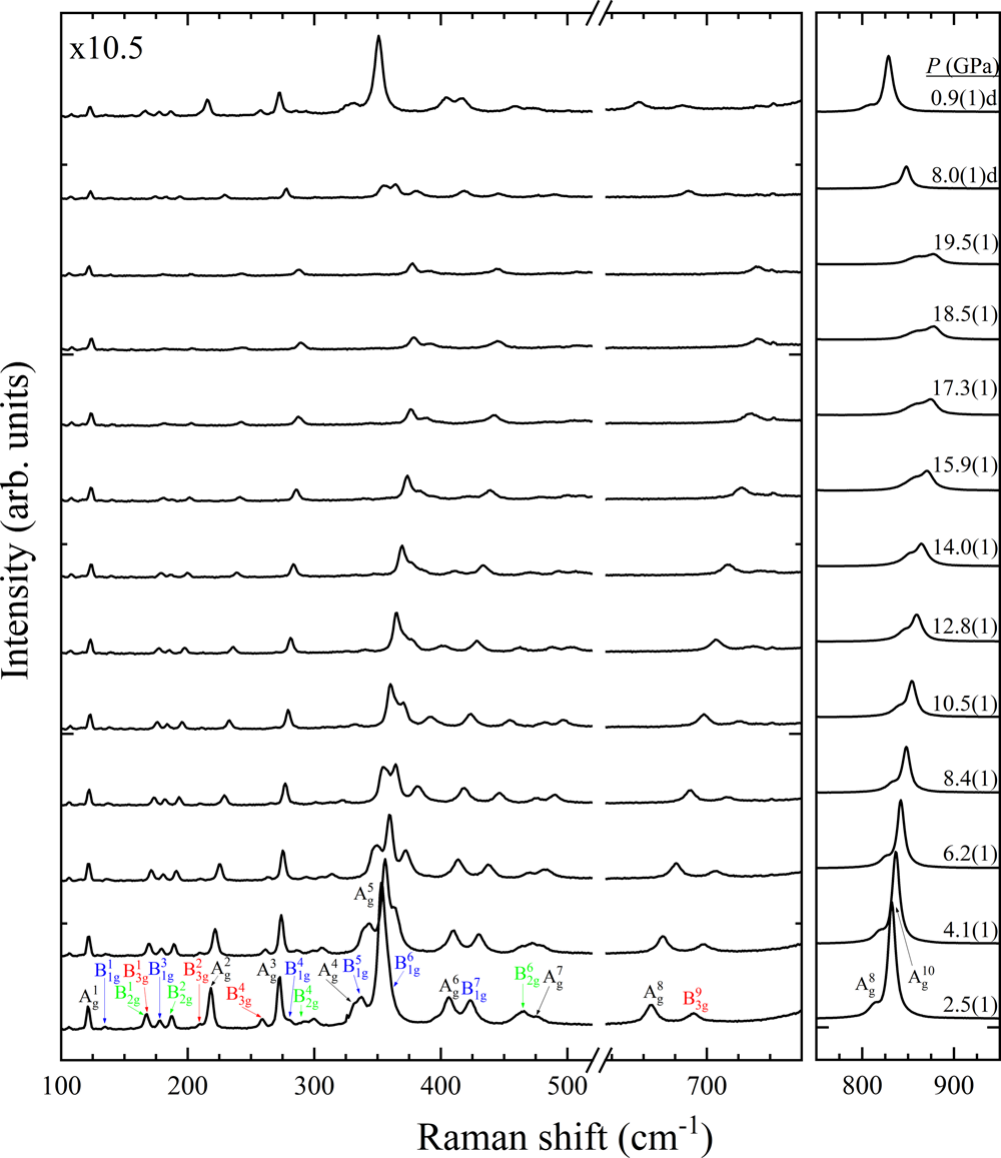
Raman spectra corresponding to Ni_3_V_2_O_8_ at selected pressures. The symmetry modes are assigned colors in the first pattern. Numbers next to the spectra indicate pressure in GPa. Downstroke data are marked with a “d”. Magnification of the first region is shown in the top left corner.

**Figure 7:**
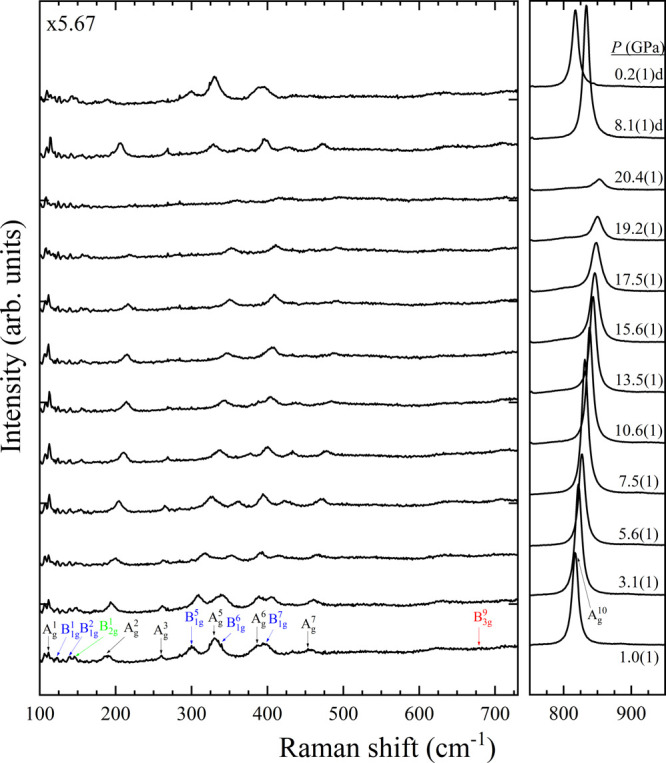
Raman spectra corresponding to Co_3_V_2_O_8_ at selected pressures. The symmetry modes are assigned colors in the first pattern. Numbers next to the spectra indicate pressure in GPa. Downstroke data are marked with a “d”. Magnification of the first region is shown in the top left corner.

In the present powder vibrational HP studies, 24 modes for Ni_3_V_2_O_8_ are monitored up to 19.5(1) GPa and 14 modes for Co_3_V_2_O_8_, up to 20.4(1) GPa, as shown for selected spectra in Figures 6 and 7, respectively. Pressure coefficients under ambient conditions are presented in Table 4. The pressure coefficients were fitted using the spectra obtained near ambient conditions, where the dependence on pressure is linear. As found in previous HP XRD studies^23^ for Ni_3_V_2_O_8_, this compound does not undergo any nonisostructural phase transition in the covered pressure range. Now, this statement can also be applied to Co_3_V_2_O_8_.

**Figure 8:**
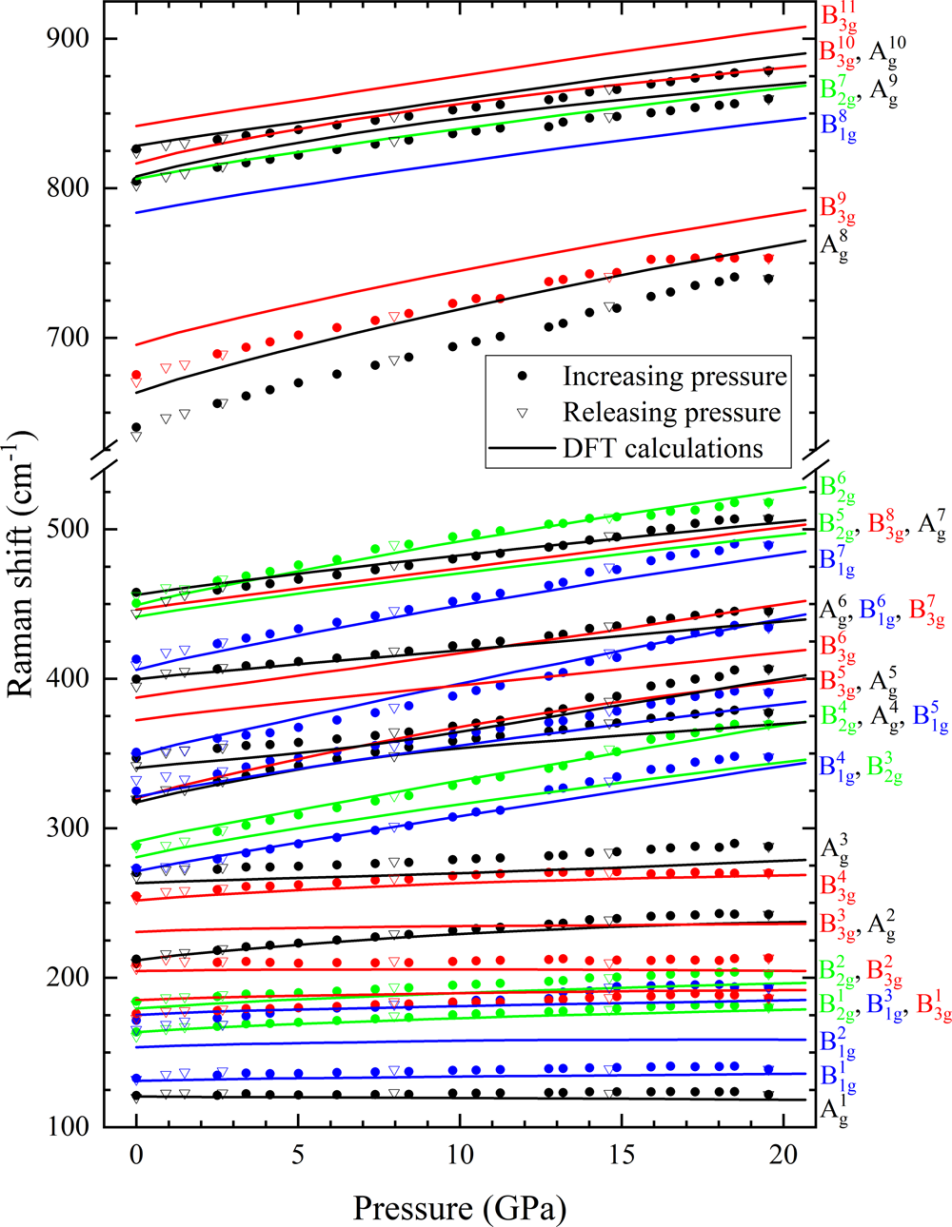
Pressure dependence of the Raman modes of Ni_3_V_2_O_8_. The symmetry modes are assigned with colors on the right, matching the end of the solid line in the figure.

**Figure 9:**
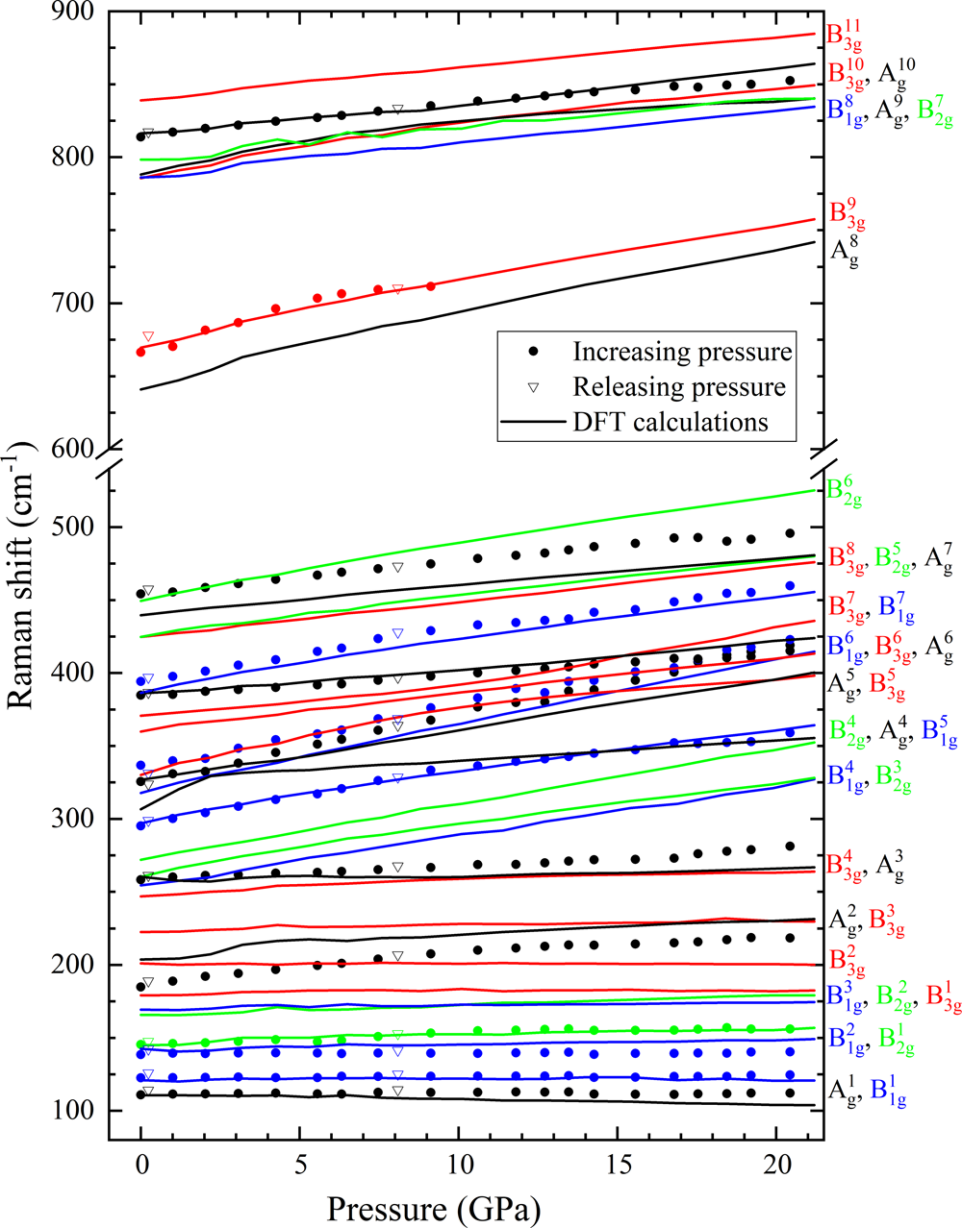
Pressure dependence of the Raman modes of Co_3_V_2_O_8_. The symmetry modes are assigned with colors on the right, matching the end of the solid line in the figure.

The pressure dependence of the calculated and experimentally measured phonon wavenumbers is shown in Figure 8 for Ni_3_V_2_O_8_ and in Figure 9 for Co_3_V_2_O_8_. It can be seen that all of the observed modes, except the A_g_^1^ mode, upshift with increasing pressure. In Co_3_V_2_O_8_, the A_g_^8^ and B_3g_^9^ modes were no longer differentiated out of the background because of signal attenuation as pressure increased. The experimental values of these coefficients are broadly in good agreement with those obtained in the ab initio calculations. The calculated lines in Figures 8 and 9 run parallel to the experimental points with the calculated lines generally shifted by less than 5% with respect to the measured data. Only a single crossover is observed experimentally between B_2g_^6^ and A_g_^7^ in Ni_3_V_2_O_8_, which is well reproduced by the DFT calculations. All data sets collected on decompression follow the same behavior as upstroke measurements. When comparing both orthovanadates, the first dissimilarity observed is that, in spite of the larger mass of Ni, all modes in Co_3_V_2_O_8_ are slightly lower in wavelength (approximately 10 cm^–1^), while high-pressure events, such as the mode crossover, occur earlier in pressure for Ni_3_V_2_O_8_ (see A_g_^6^-B_1g_^7^, A_g_^3^-B_1g_^4^, or A_g_^6^-B_3g_^7^ in Figures 8 and 9). These observations suggest that Ni_3_V_2_O_8_ behaves as a pressurized version of Co_3_V_2_O_8_.

### 3.5 High-Pressure X-ray Diffraction (Co_3_V_2_O_8_)

**Figure 10:**
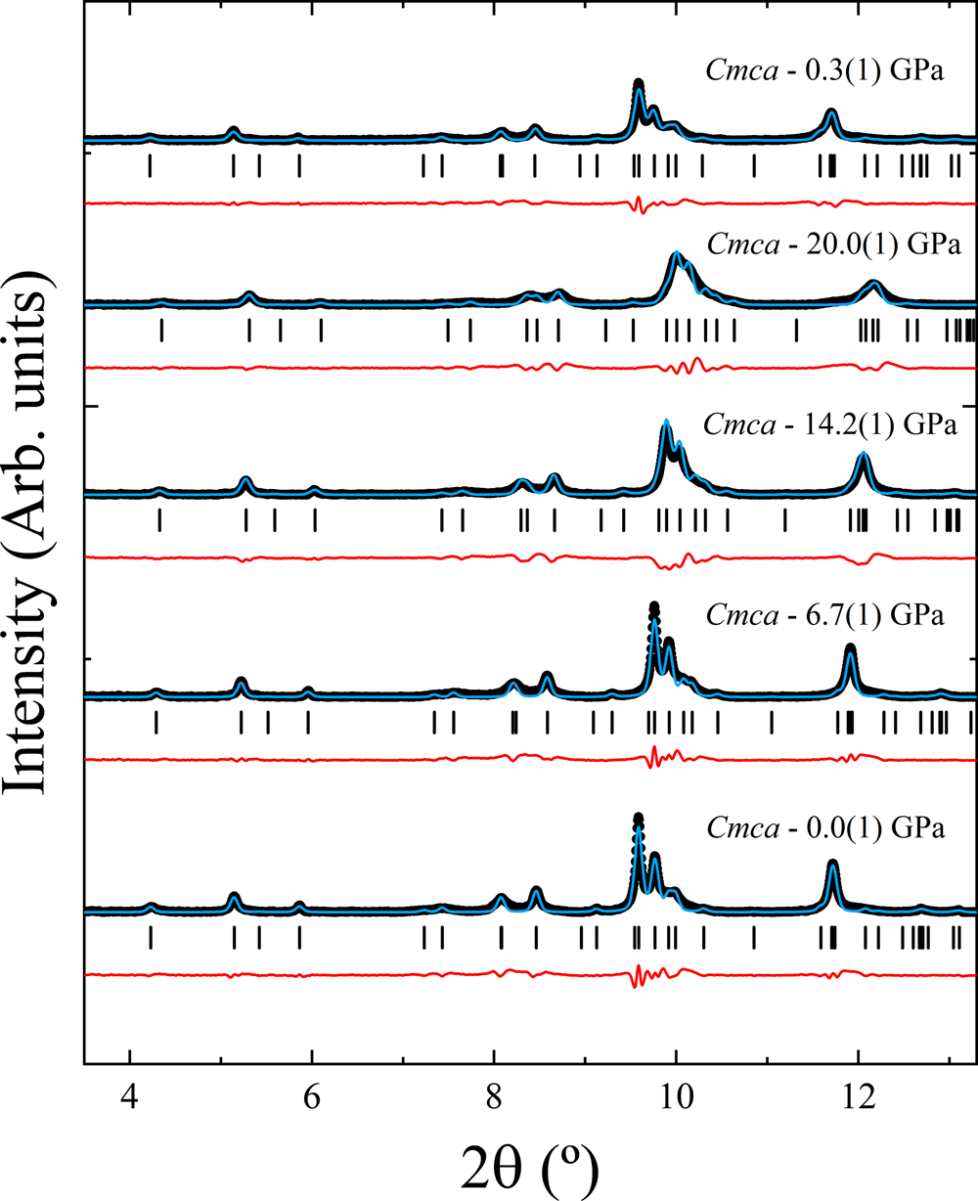
XRD patterns at selected pressures (black dots) of Co_3_V_2_O_8_. Le Bail fits and residuals are shown with blue and red lines, respectively. Ticks indicate the Bragg peaks for the corresponding structural phase. Pressures are indicated in the figure. The top trace corresponds to the last experiment made during decompression.

Using the XRD patterns collected for powder Co_3_V_2_O_8_ under HP, the orthorhombic structure (space group *Cmca*, number 64) was fitted from 0.0(1) to 20.0(1) GPa. Le Bail refinement^55^ results are shown in Figure 10 for selected pressures. The patterns shown correspond to positions of the DAC where there was no Cu signal. This compound does not exhibit any phase transition in the mentioned pressure region, as was also published for Ni_3_V_2_O_8_.^24^

**Figure 11:**
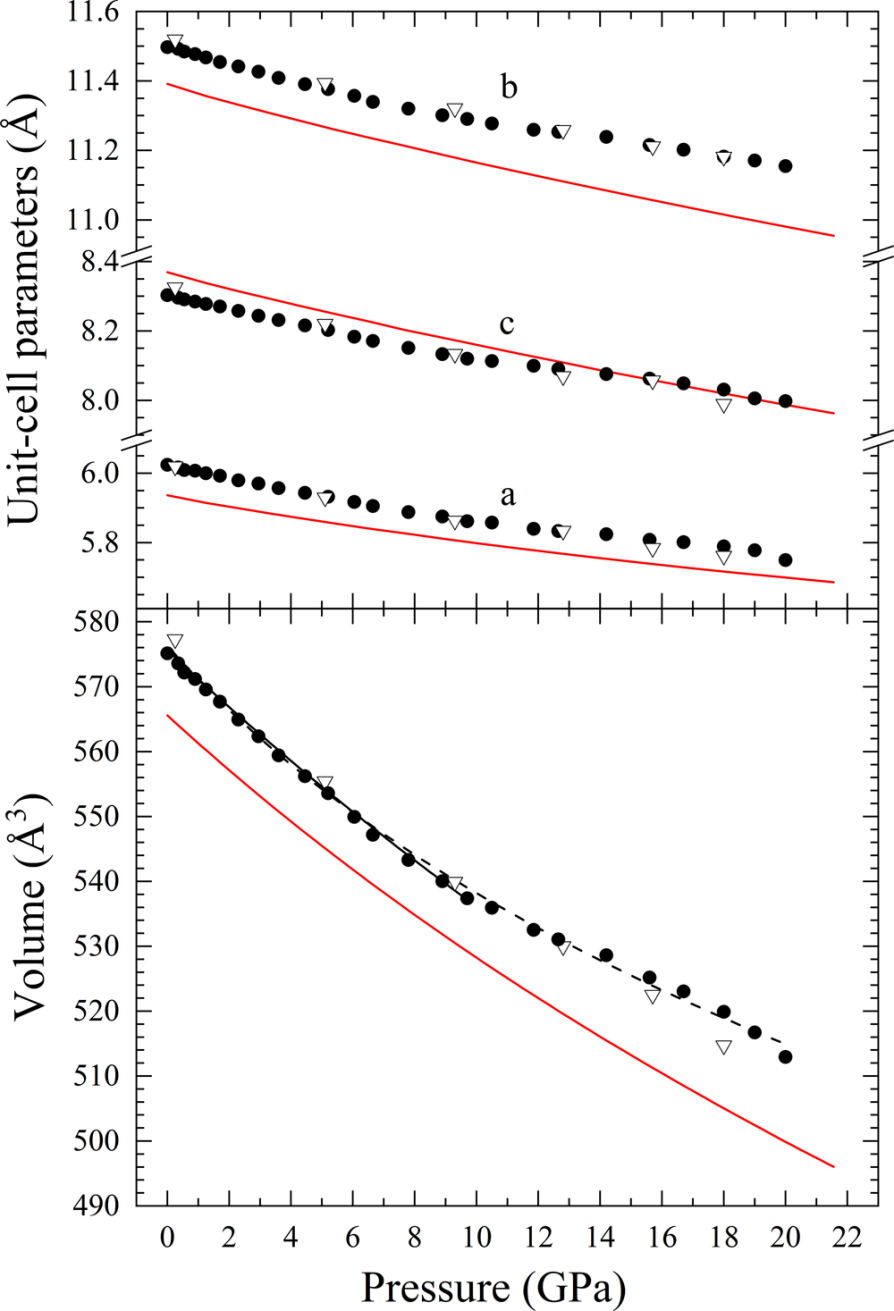
Pressure dependence of the unit-cell parameters (top) and volume (bottom) of Co_3_V_2_O_8_. Black symbols represent experimental measurements, and red lines are DFT calculations. Full circles represent upward pressure, while empty triangles release pressure data. The solid black line and dashed black line are the Birch–Murnaghan EOS fitting of the experimental unit-cell volume up to 9.7 and 20 GPa, respectively.

Subsequently, the pressure dependence of the unit-cell parameters and corresponding volume of the orthorhombic structure of Co_3_V_2_O_8_ is reported in Figure 11, using the results from the Le Bail fits^55^ and peak indexation with UNITCELL.^56^ At 10.5(1) GPa, a slight change in the evolution of all three unit-cell axes was noticed. This fact coincides with the end of the hydrostatic region of the ME pressure-transmitting media,^27^ which is probably the reason that the linear compressibility of each axis is reduced. This nonhydrostatic effect led us to report two separate equations of state (EOSs), one up to the hydrostatic limit of ME (9.3(1) GPa) and another up to the maximum pressure (20.0(1) GPa). Thus, the unit-cell volume was fitted using a third-order Birch–Murnaghan EOS^48^ employing EosFit7 software.^57^ The third order of the EOS was determined from the Eulerian strain-normalized pressure dependence of the data.^58^ All EOS parameters are reported in Table 5, along with literature ones for other Kagomé-staircase orthovanadates. The unit-cell parameters obtained by DFT calculations differ from the experimental parameters by approximately 1% in terms of absolute value. Furthermore, the bulk moduli obtained in the EOS for both calculations (129.2(7) GPa) and experiments up to 9.3(1) GPa (127.4(4) GPa) are in good agreement. Comparing these results with the bulk modulus reported for Ni_3_V_2_O_8_,^24^ it can be noticed that Co orthovanadate is more compressible than Ni orthovanadate. For this comparison, the two bulk moduli obtained by a second order EOS and under hydrostatic conditions were used, whose values are 122(4) and 143(3) GPa for Co and Ni vanadates, respectively (see Table 5). This fact agrees with the observations reported in the HP Raman section (3.4), where it was concluded that Ni_3_V_2_O_8_ behaves as a pressurized version of Co_3_V_2_O_8_. Overall, Ni and Co vanadates show bulk moduli within the range of all other Kagomé-staircase orthovanadates, with Mg_3_V_2_O_8_ being the highest (152(4) GPa)^23^ and Mn_3_V_2_O_8_, the lowest (106(3) GPa)^21^ to date.

**Table 5:** EOS Parameters (Cell Volume per Formula Unit, Bulk Modulus, and Its First Derivative) for Reported Kagomé-Staircase Orthovanadates

XRD HP experiments	*V*_0_ (Å^3^)	*B*_0_ (GPa)	*B*_0_′
Co_3_V_2_O_8_ up to 9.3(1) GPa (this work)	575.6(3)	127(3)	2.8 (9)
	575.9(2)	122(4)	4.0 (fixed)
Co_3_V_2_O_8_ up to 20.0(1) GPa (this work)	576.3(8)	106(7)	9.7(1.3)
	574.0(6)	142(3)	4.0 (fixed)
Ni_3_V_2_O_8_ up to 7.6(1) GPa^24^	555.7(2)	139(3)	4.4(3)
	555.3(2)	143(3)	4.0 (fixed)
Mn_3_V_2_O_8_ up to 12 GPa^21^	623.4(2)	116(3)	2.6(5)
	624.5(5)	106(3)	4.0 (fixed)
Zn_3_V_2_O_8_ up to 15 GPa^22^	585.0(4)	115(2)	5.1(6)
	585.1(1)	120(2)	4.0 (fixed)
Mg_3_V_2_O_8_ up to 17 GPa^23^	576.15(3)	141(3)	5.9(8)
	576.15(3)	152(4)	4 (fixed)

DFT calculations	V_0_ (Å^3^)	B_0_ (GPa)	B_0_′
Co_3_V_2_O_8_ (this work)	565.6(3)	129(7)	3.7(4)
	565.9(6)	126(6)	4.0 (fixed)
Ni_3_V_2_O_8_^59^	527.9	171	4.5
Zn_3_V_2_O_8_^59^	563.2	136	5.4
Mg_3_V_2_O_8_^59^	539.7	146	4.4

From the reported unit-cell parameters, the linear isothermal compressibility was calculated for all axes of the orthorhombic structure: , where *x* = *a*, *b*, or *c*. The linear compressibilities obtained are κ_*a*_ = 2.73(15) × 10^–3^ GPa^–1^, κ_*b*_ = 1.98(4) × 10^–3^ GPa^–1^, and κ_*c*_ = 2.29(14) × 10^–3^ GPa^–1^. The region used for the fits is from 0.0(1) to 9.3(1) GPa to guarantee that only hydrostatic data are used. When these compressibilities are compared with the experimentally obtained ones for Ni_3_V_2_O_8_^23^ and simulated for other orthovanadates,^59^ it can be clearly appreciated that they follow the same behavior followed for this family of compounds, but the *b*-axis of Co_3_V_2_O_8_ is slightly more compressible. Once more, this can be related to the “compressed” structure of Ni_3_V_2_O_8_ indicated in Section 3.4, since the *b*-axis is mainly influenced by the layers of CoO_6_ octahedra (see Figure 1), which are more compressible than the NiO_6_ octahedra. The DFT-calculated change in bond distances within the covered pressure range can be seen in Figures S1 and S2 for both compounds.

## 4 Conclusions

High-pressure vibrational studies were performed for Ni_3_V_2_O_8_ and Co_3_V_2_O_8_ powders up to 19.5(1) and 20.4(1) GPa, respectively, and no phase transition was found. Polarized Raman and infrared measurements on single crystals were used to separate and identify the symmetry of the vibrational modes for both compounds under ambient conditions. Ab initio DFT calculations are reported to confirm the symmetry, ambient pressure wavenumber value, and pressure coefficients of all the experimental modes found (24 for Ni_3_V_2_O_8_ and 17 for Co_3_V_2_O_8_ out of the 36 Raman active modes). Although both Ni and Co orthovanadates present a similar vibrational behavior under pressure, it is found that Ni_3_V_2_O_8_ exhibits a more compact version of the structure. HP angle dispersive powder XRD analysis up to 20.0(1) GPa was also performed for Co_3_V_2_O_8_. Anisotropic compressibility and EOS parameters (including bulk moduli) are obtained from both the experimental results and the DFT calculations. Excellent agreement is found between the two sets of data.

## Data Availability

The data that support the findings of this study are available from the corresponding author upon reasonable request.

## Supporting Information Available

Atomic motions of the acoustic and infrared active modes, the Raman active modes, and the silent modes; DFT calculated bond distances (PDF)
