# Supplementary material for: High-Pressure Vibrational and Structural Properties of Ni3V2O8 and Co3V2O8 up to 20 GPa
Source: J Phys Chem C Nanomater Interfaces. 2023 Oct 30;127(44):21684–94. doi: 10.1021/acs.jpcc.3c04019 (PMC10851775; doi:10.1021/acs.jpcc.3c04019)
Supplement: Supplementary file 1 — jp3c04019_si_001.pdf [file jp3c04019_si_001.pdf]

Supporting Information

High-Pressure Vibrational and Structural Properties of $Ni_3V_2O_8$ and $Co_3V_2O_8$ up to 20 GPa

Josu Sánchez-Martín[1], Julio Pellicer-Porres[1], Akun Liang[1,2], Jordi Ibáñez[3], Robert Oliva[3], Catalin Popescu[4], Zhangzhen He[5], Plácida Rodríguez-Hernández[6], Alfonso Muñoz[6], Daniel Errandonea[1]

[1] Departamento de Física Aplicada-ICMUV, MALTA-Consolider Team, Universidad de Valencia, Dr. Moliner 50, Burjassot, 46100 Valencia, Spain

[2] CSEC, The University of Edinburgh, UoE, School of Physics and Astronomy, Edinburgh U.K.

[3] Geosciences Barcelona (GEO3BCN), MALTA-Consolider Team, CSIC, Lluís Solé i Sabarís s/n, 08028 Barcelona, Spain

[4] CELLS-ALBA Synchrotron Light Facility, MALTA-Consolider Team, Cerdanyola del Vallès, 08290 Barcelona, Spain

[5] State Key Laboratory of Structural Chemistry, Fujian Institute of Research on the Structure of Matter, Chinese Academy of Sciences, Fuzhou, Fujian 350002, China

[6] Departamento de Física, MALTA-Consolider Team, Instituto de Materiales y Nanotecnología, Universidad de La Laguna, San Cristóbal de La Laguna, E-38200 Tenerife, Spain

* E-mail: josu.sanchez@uv.es

Table S1. Atomic motions of the acoustic (first B_{1u}, B_{2u} and B_{3u}) and infrared active modes of $M_3V_2O_8$ (M = Ni, Co)*. The black vectors were calculated with VASP using the primitive unit-cell and then represented with Jmol. M is in pink, V in gray and O in red.

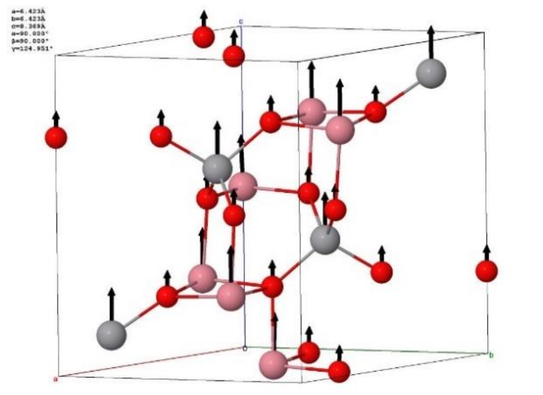 B_{1u}^{1} (acoustic)	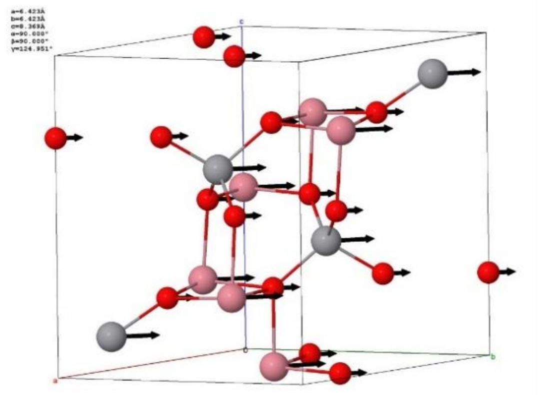 B_{2u}^{1} (acoustic)
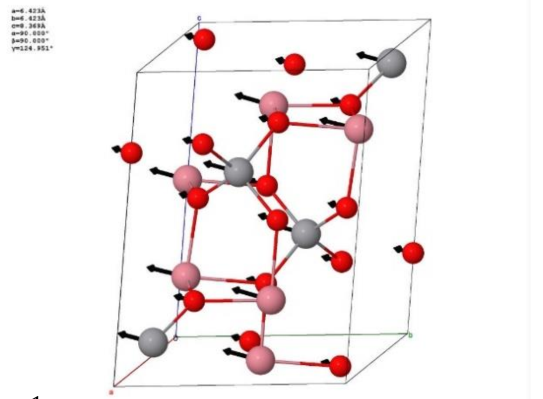 B_{3u}^{1} (acoustic)	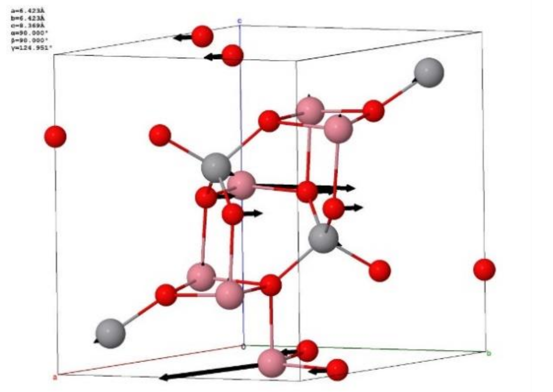 B_{1u}^{2} (124 cm^{-1})
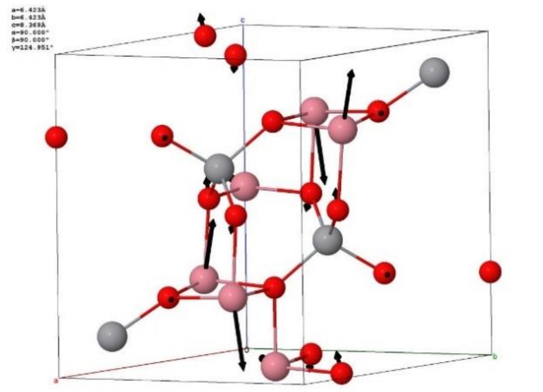 B_{3u}^{2} (144 cm^{-1})	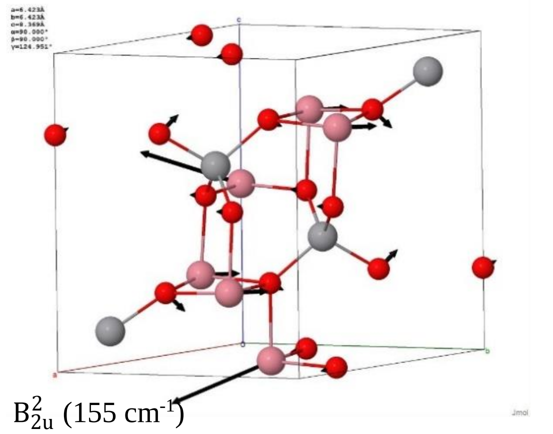 B_{2u}^{2} (155 cm^{-1})

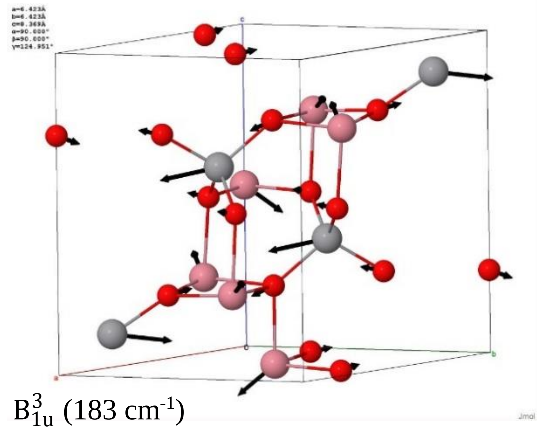

B_{1u}^{3} (183 cm^{-1})

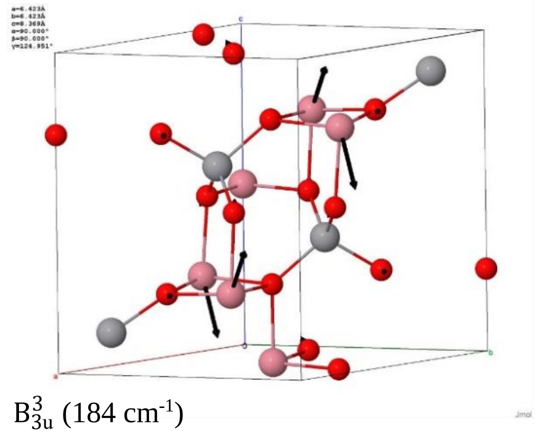

B_{3u}^{3} (184 cm^{-1})

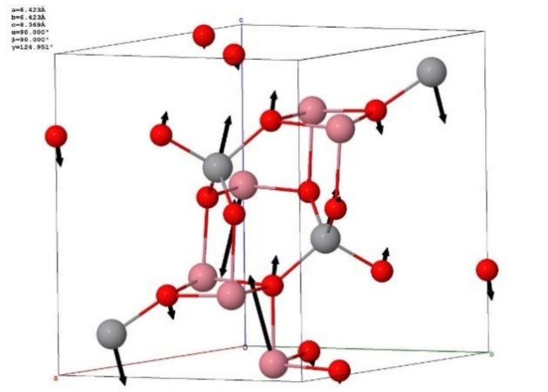

B_{2u}^{3} (182 cm^{-1})

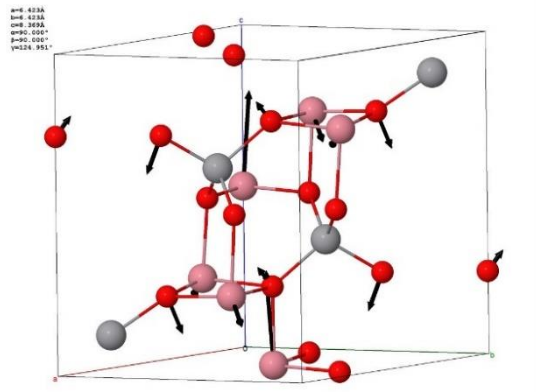

B_{1u}^{4} (200 cm^{-1})

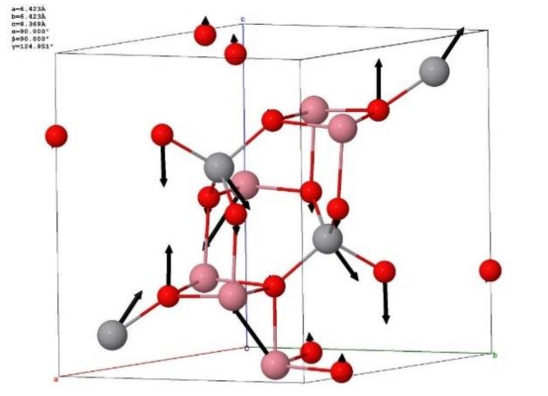

B_{2u}^{4} (209 cm^{-1})

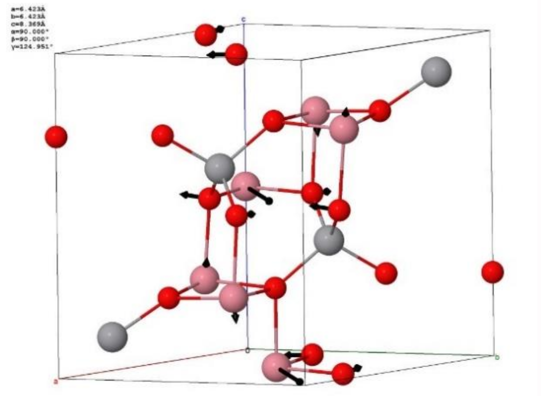

B_{3u}^{4} (245 cm^{-1})

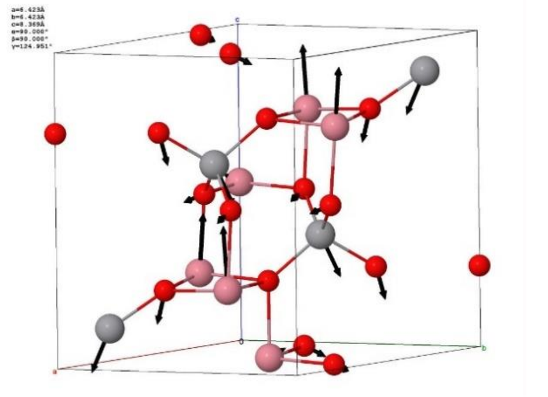

B_{1u}^{5} (247 cm^{-1})

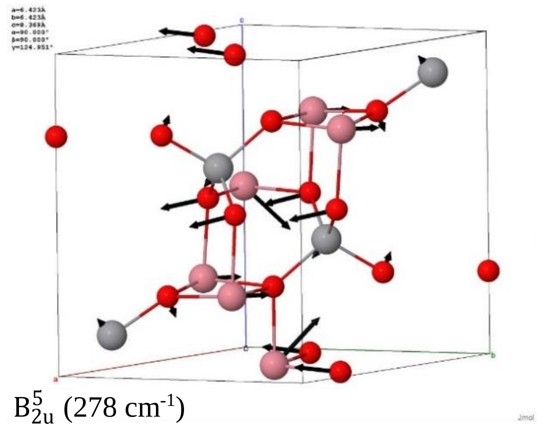

B_{2u}^{5} (278 cm^{-1})

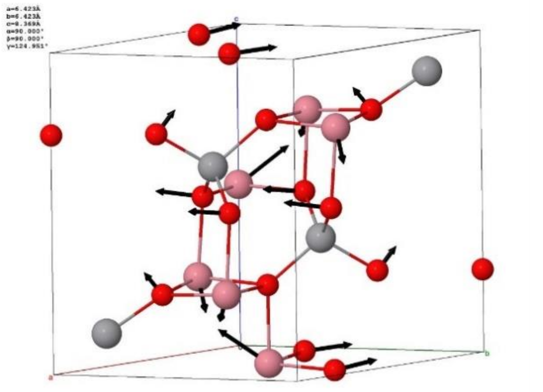

B_{1u}^{6} (285 cm^{-1})

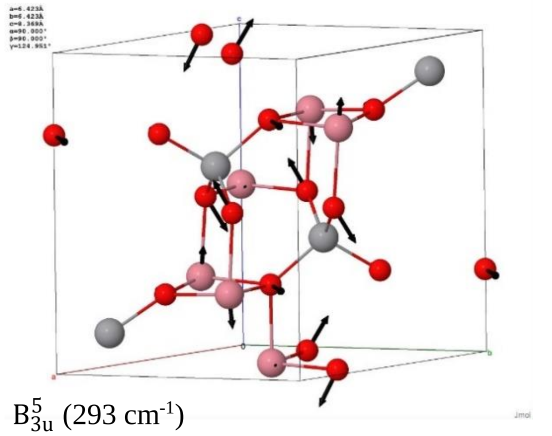

B_{3u}^{5} (293 cm^{-1})

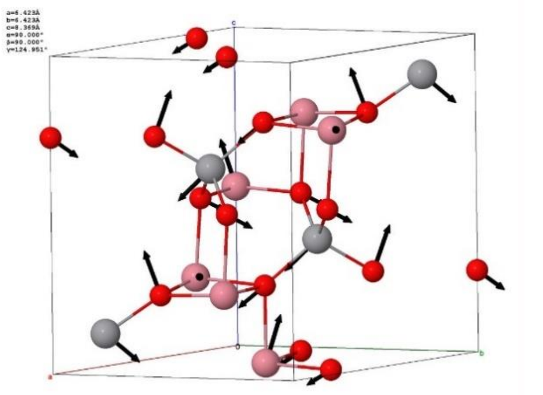

B_{1u}^{7} (305 cm^{-1})

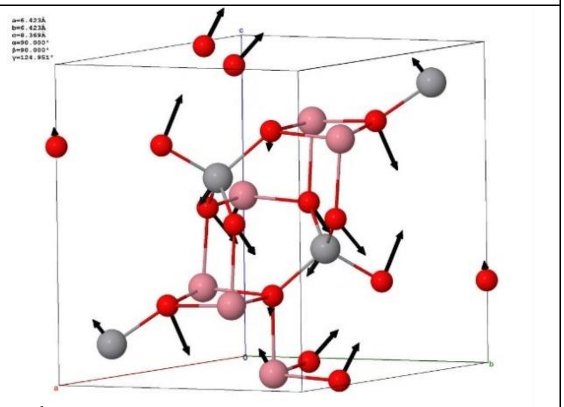

B_{2u}^{6} (301 cm^{-1})

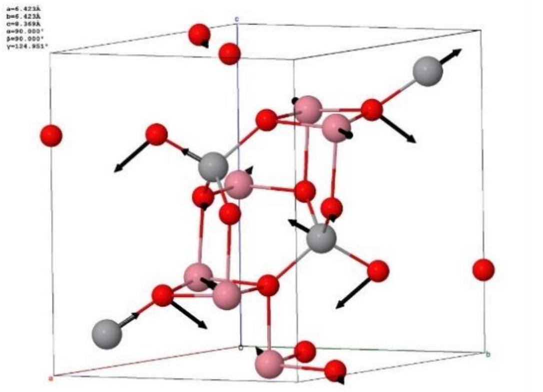

B_{1u}^{8} (332 cm^{-1})

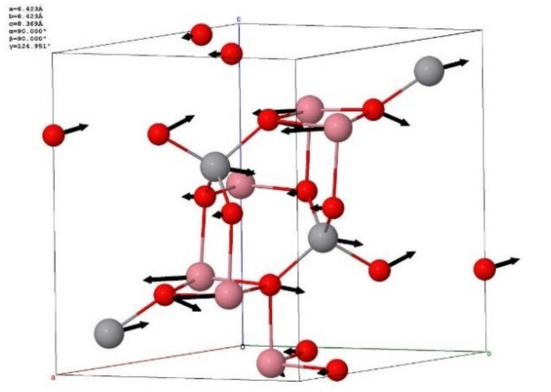

B_{2u}^{7} (320 cm^{-1})

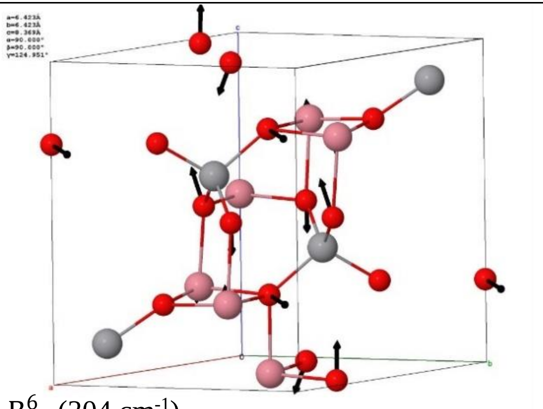

B_{3u}^{6} (304 cm^{-1})

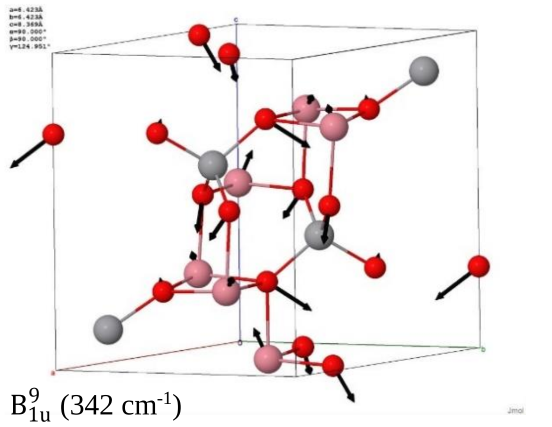

B_{1u}^{9} (342 cm^{-1})

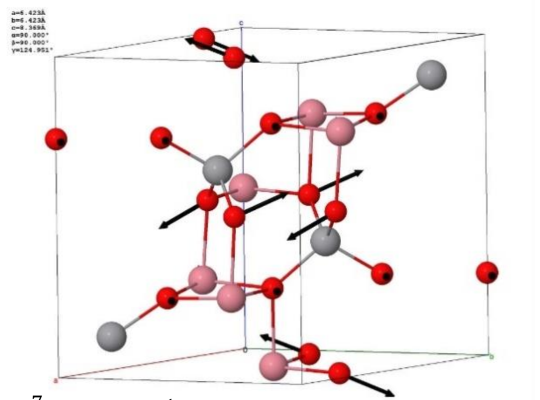

B_{3u}^{7} (353 cm^{-1})

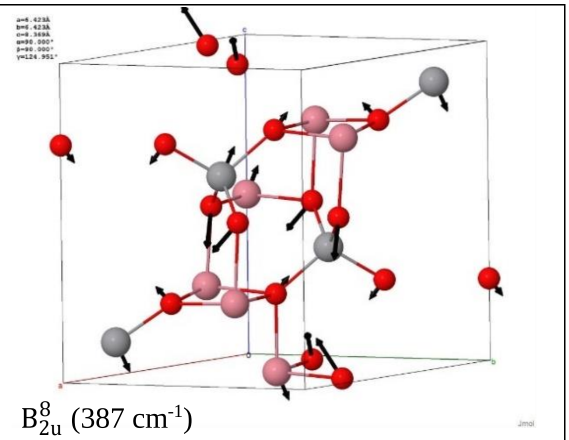

B_{2u}^{8} (387 cm^{-1})

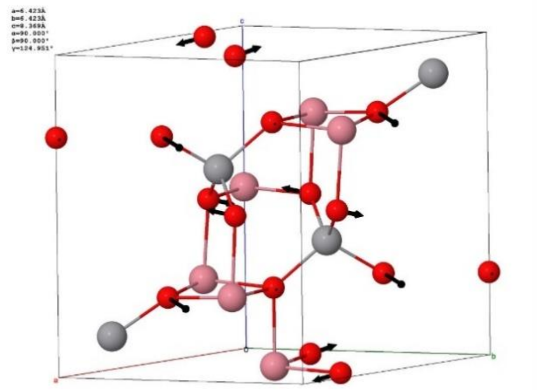

B_{3u}^{8} (396 cm^{-1})

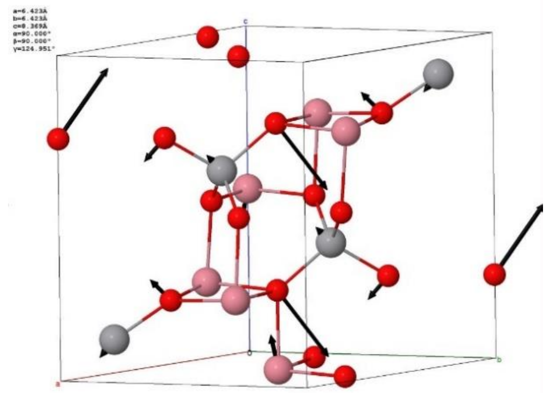

B_{2u}^{9} (421 cm^{-1})

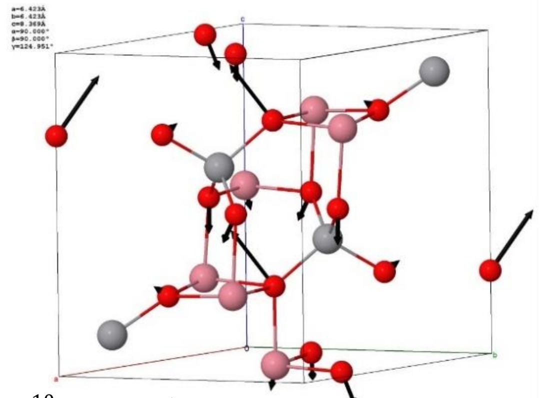

B_{1u}^{10} (427 cm^{-1})

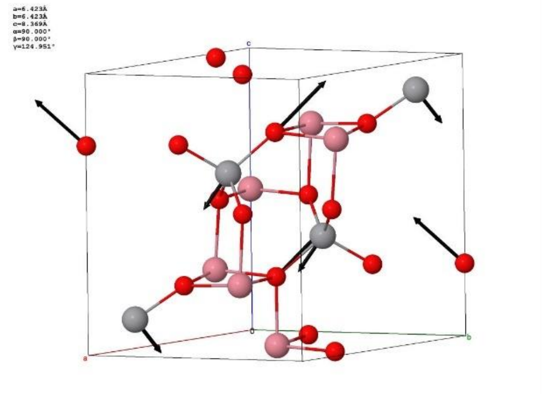

B_{1u}^{11} (636 cm^{-1})

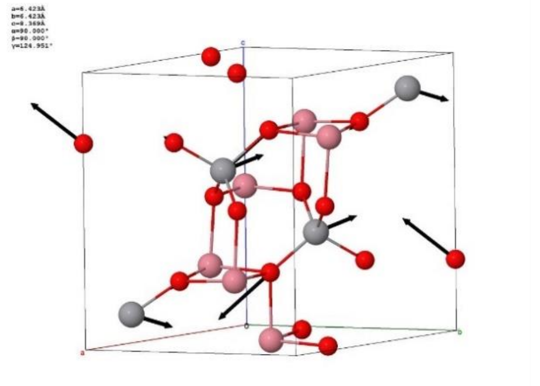

B_{2u}^{10} (642 cm^{-1})

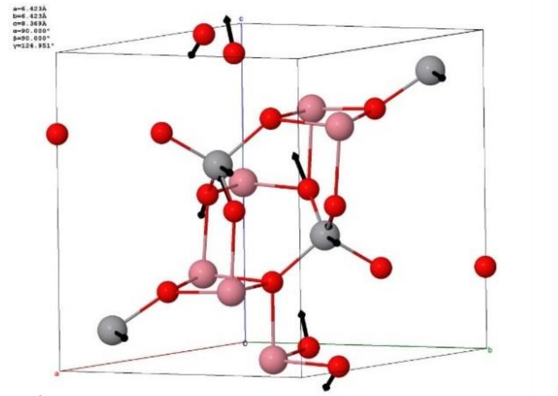

B_{3u}^{9} (806 cm^{-1})

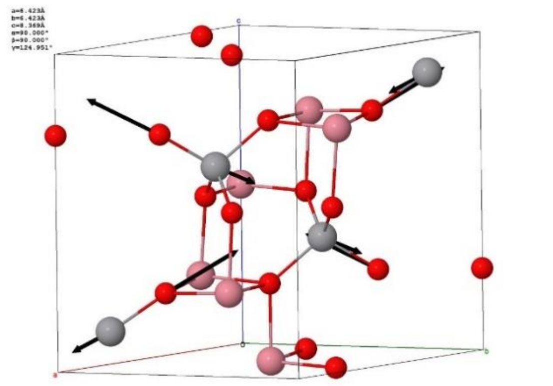 B_{1u}^{12} (770 cm^{-1})	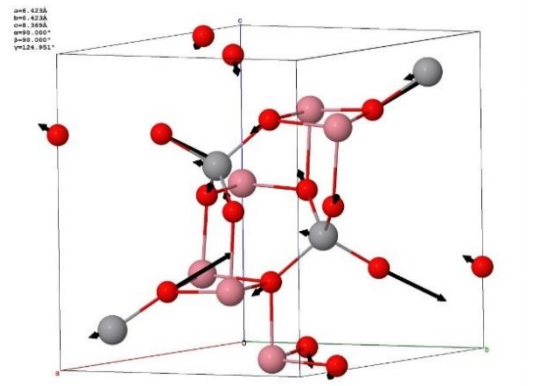 B_{2u}^{11} (790 cm^{-1})
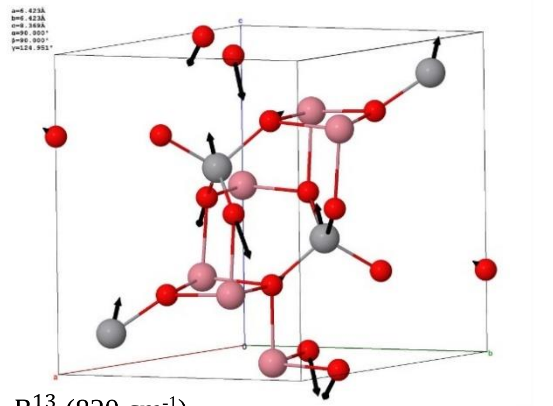 B_{1u}^{13} (820 cm^{-1})	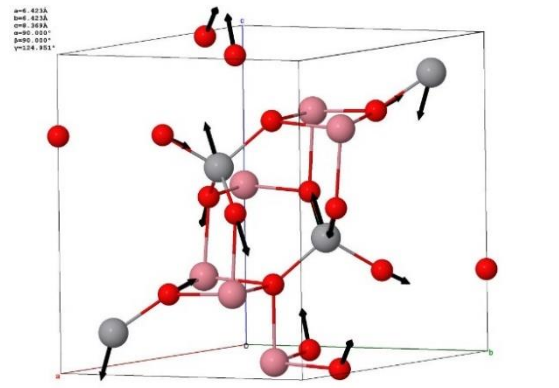 B_{2u}^{12} (864 cm^{-1})

* Vibration amplitudes vary with the particular cation, but the direction does not. In this table, Co is used.

Table S2. Atomic motions of the Raman active modes of $M_3V_2O_8$ (M = Ni, Co)*. The black vectors were calculated with VASP using the primitive unit-cell and then represented with Jmol. M is in pink, V in gray and O in red.

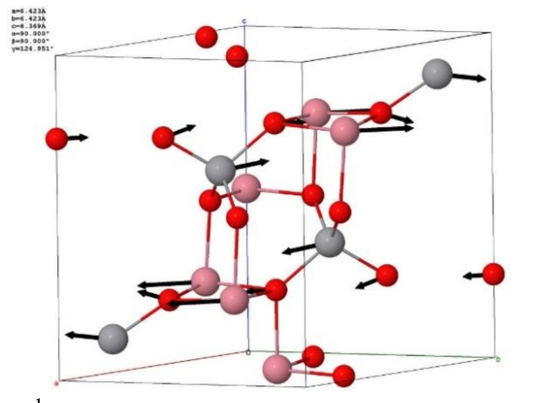 A_{g}^{1} (111 cm^{-1})	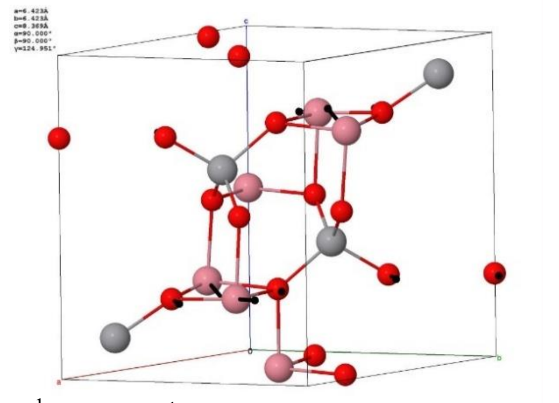 B_{1g}^{1} (121 cm^{-1})

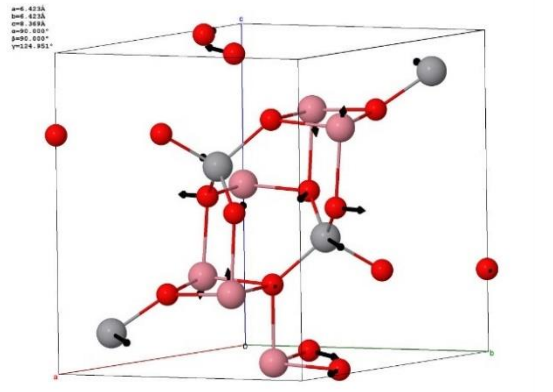

B_{1g}^{2} (143 cm^{-1})

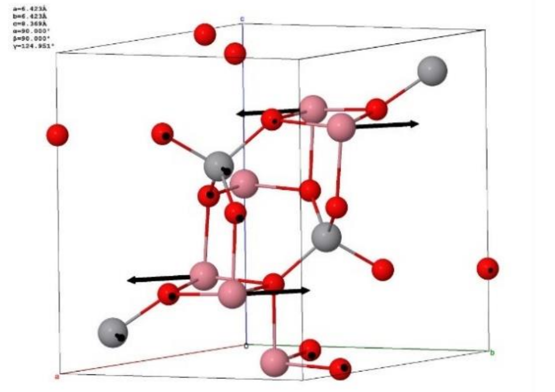

B_{2g}^{1} (145 cm^{-1})

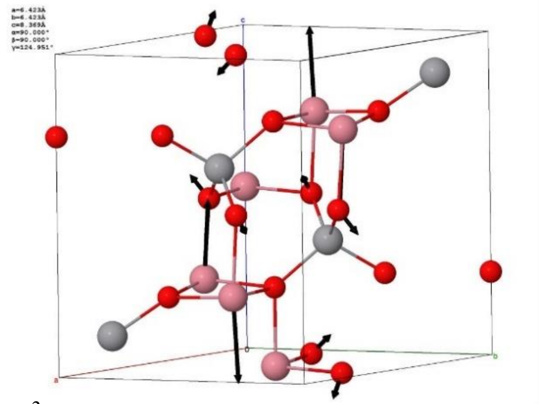

B_{1g}^{3} (169 cm^{-1})

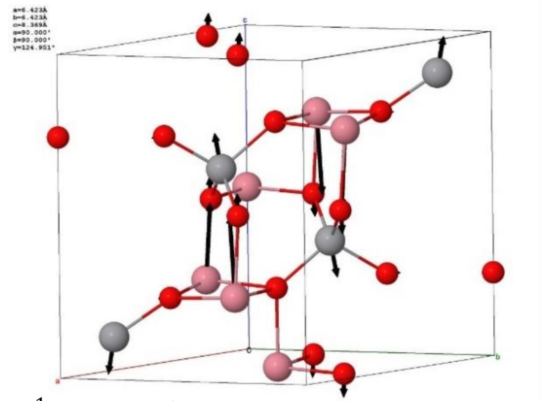

B_{3g}^{1} (179 cm^{-1})

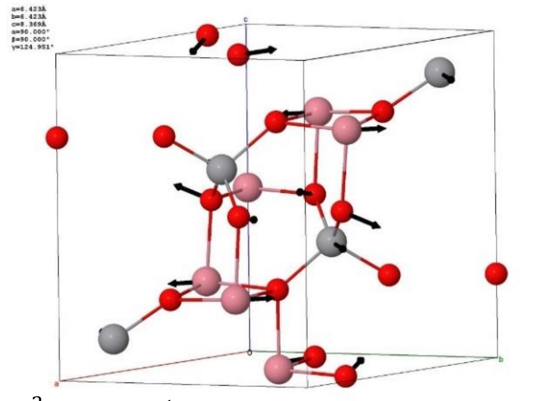

B_{2g}^{2} (166 cm^{-1})

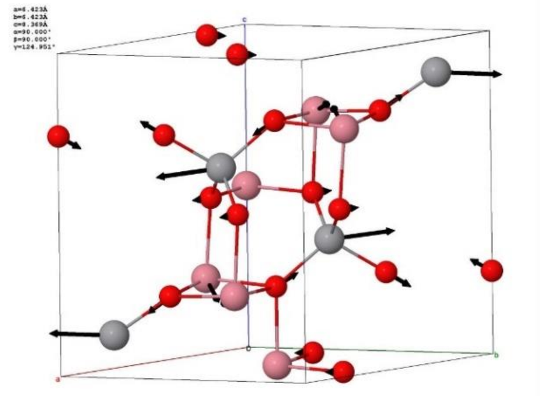

B_{3g}^{2} (201 cm^{-1})

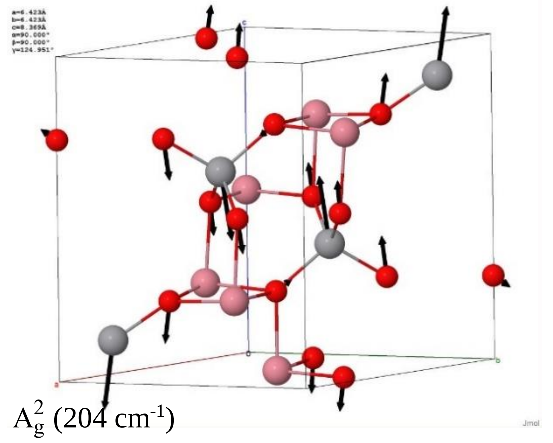

A_{g}^{2} (204 cm^{-1})

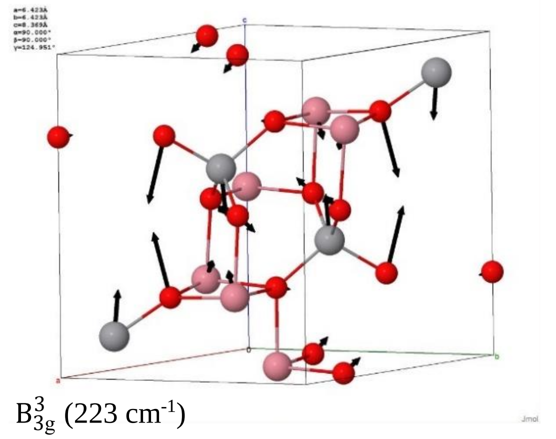

B_{3g}^{3} (223 cm^{-1})

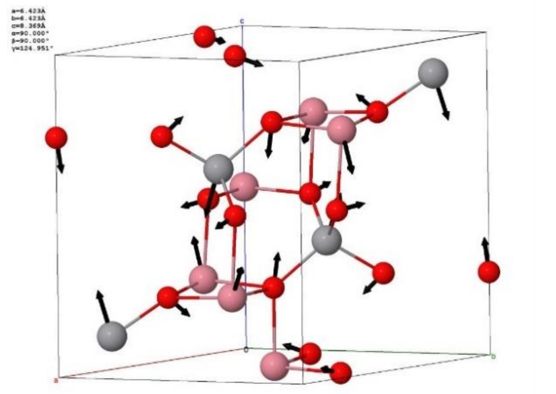 B_{3g}^{4} (247 cm^{-1})	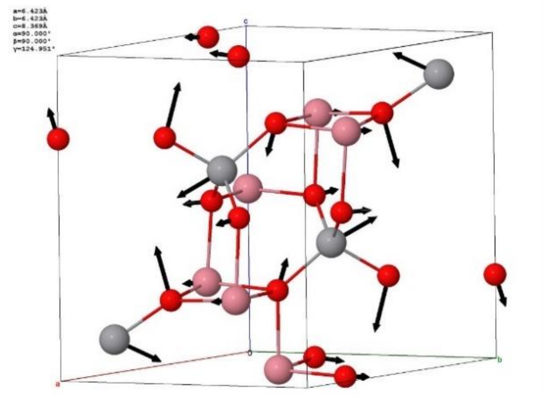 A_{g}^{3} (260 cm^{-1})
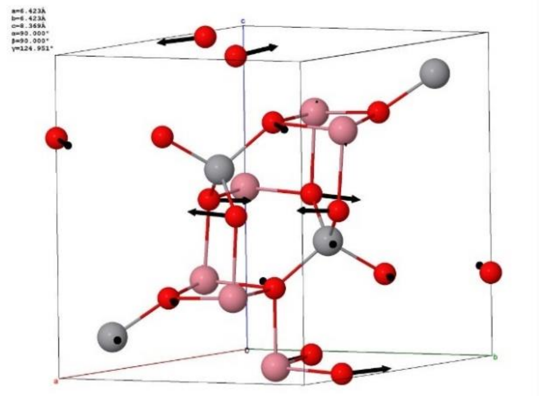 B_{1g}^{4} (254 cm^{-1})	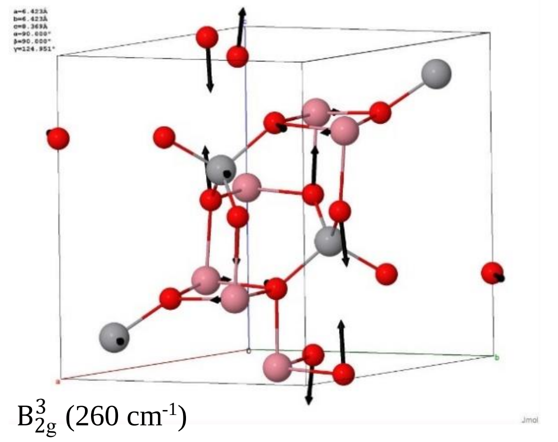 B_{2g}^{3} (260 cm^{-1})
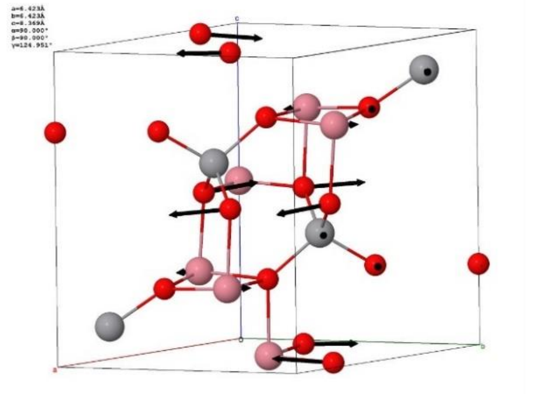 B_{2g}^{4} (272 cm^{-1})	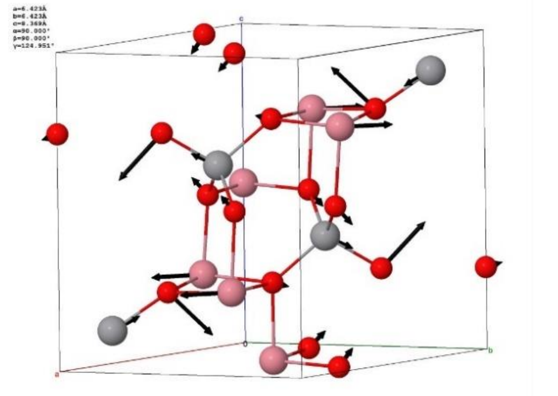 A_{g}^{4} (306 cm^{-1})
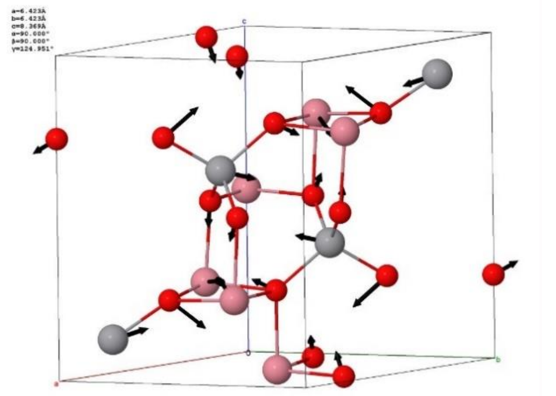 B_{3g}^{5} (330 cm^{-1})	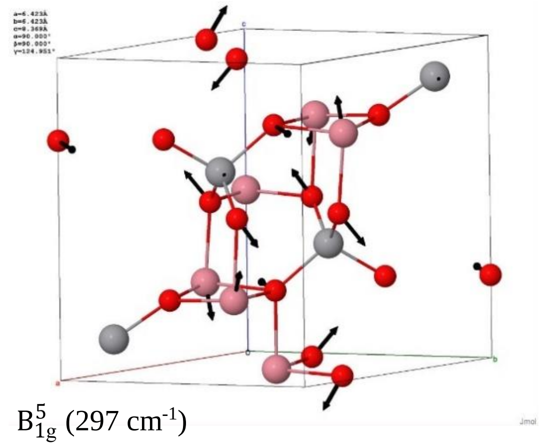 B_{1g}^{5} (297 cm^{-1})

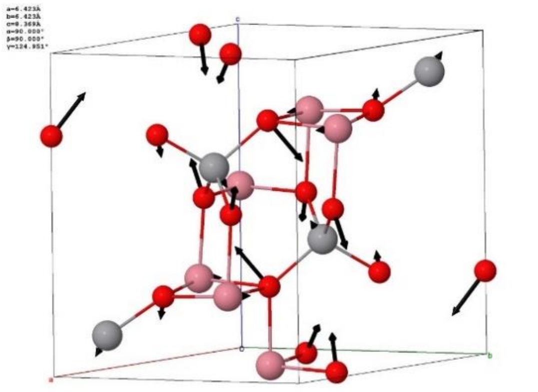 A_g^5 (327 cm^{-1})	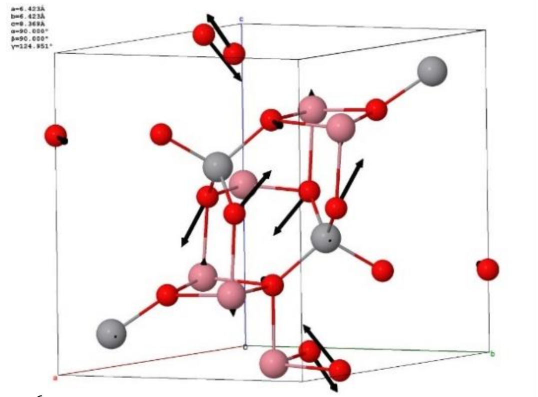 B_{1g}^6 (318 cm^{-1})
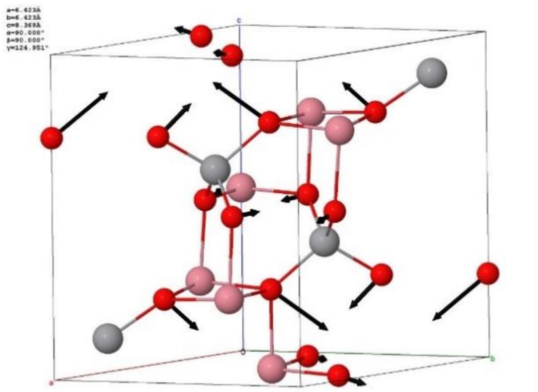 B_{3g}^6 (360 cm^{-1})	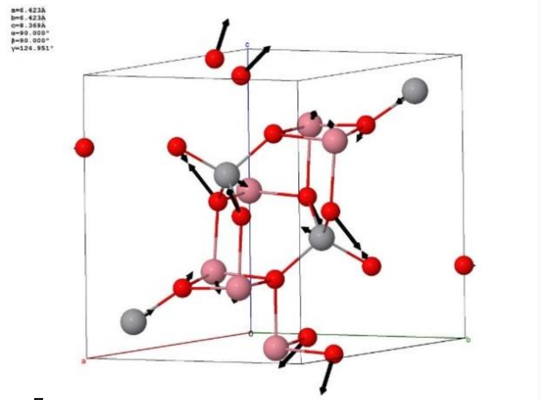 B_{3g}^7 (371 cm^{-1})
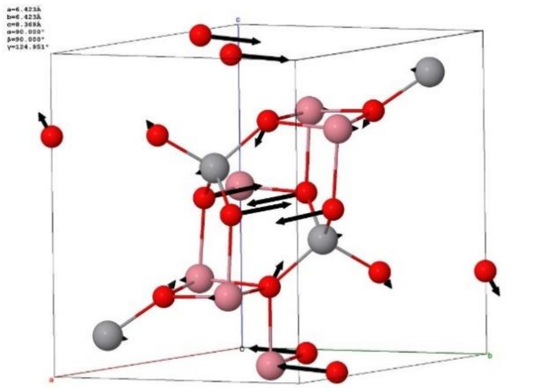 A_g^6 (386 cm^{-1})	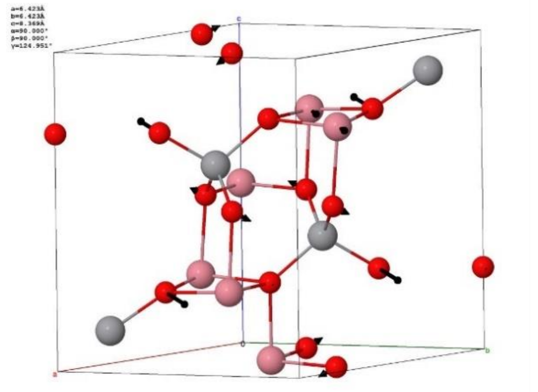 B_{1g}^7 (386 cm^{-1})
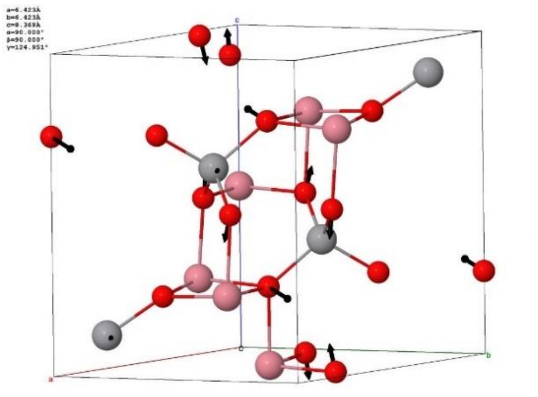 B_{2g}^5 (425 cm^{-1})	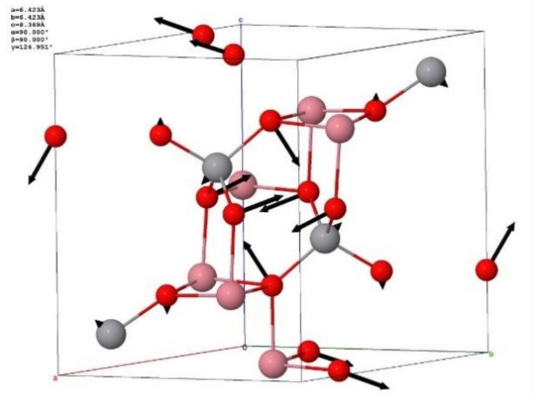 B_{3g}^8 (425 cm^{-1})

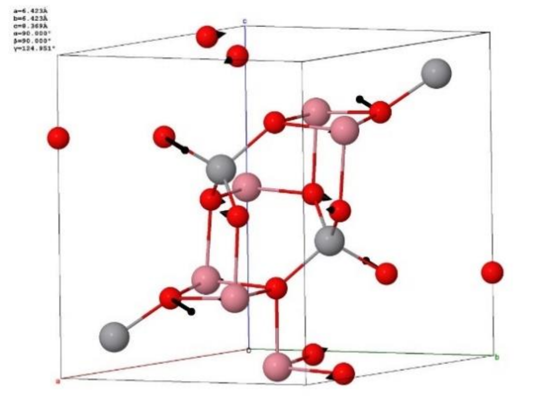

B_{2g}^{6} (449 cm^{-1})

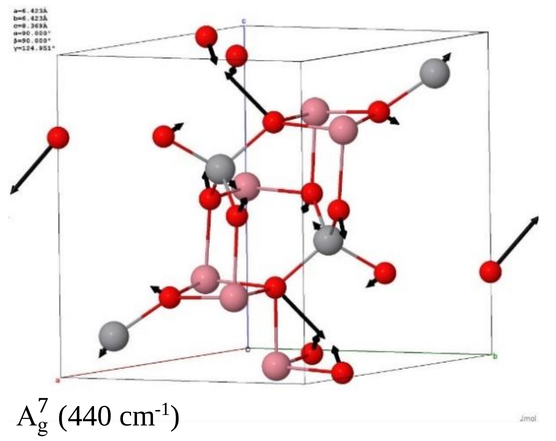

A_{g}^{7} (440 cm^{-1})

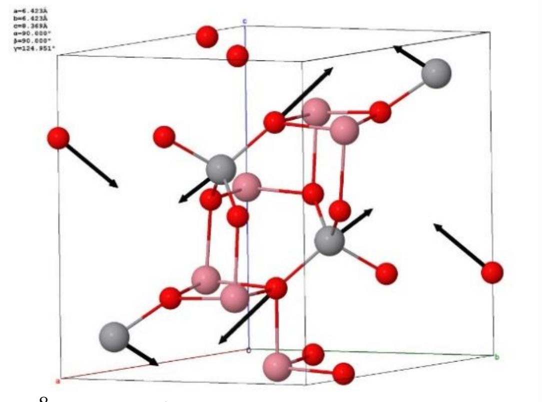

A_{g}^{8} (642 cm^{-1})

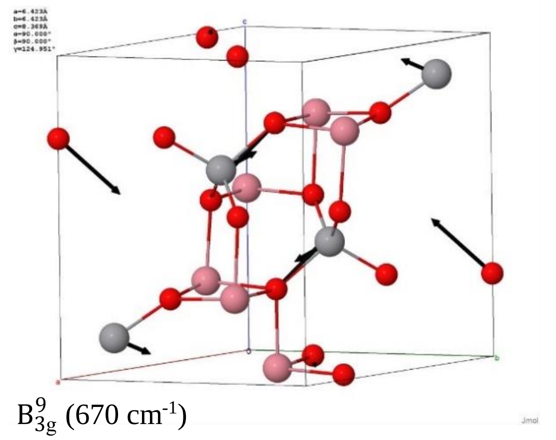

B_{3g}^{9} (670 cm^{-1})

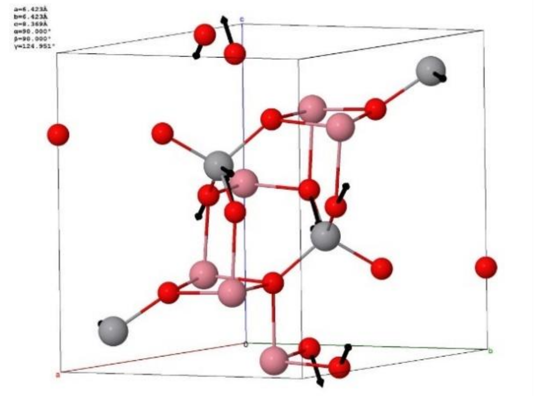

B_{1g}^{8} (786 cm^{-1})

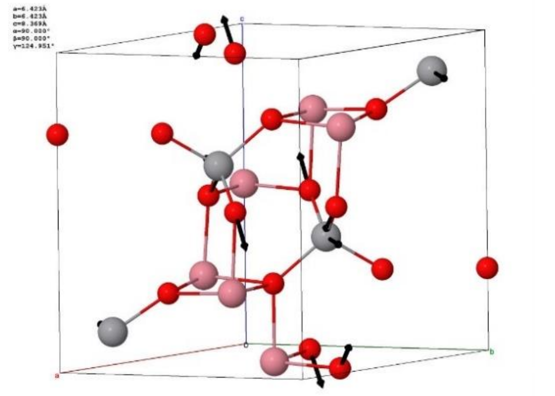

B_{2g}^{7} (798 cm^{-1})

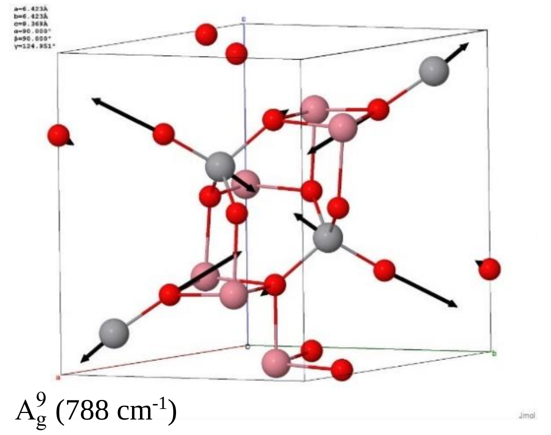

A_{g}^{9} (788 cm^{-1})

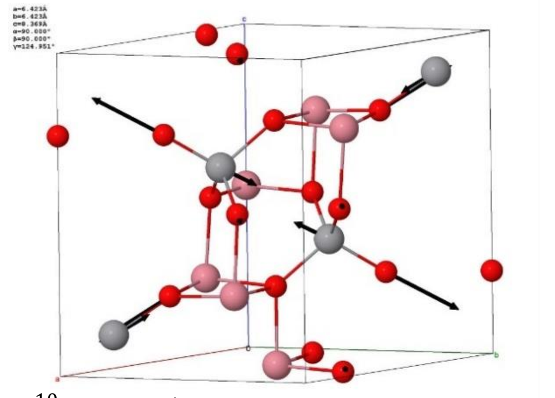

B_{3g}^{10} (786 cm^{-1})

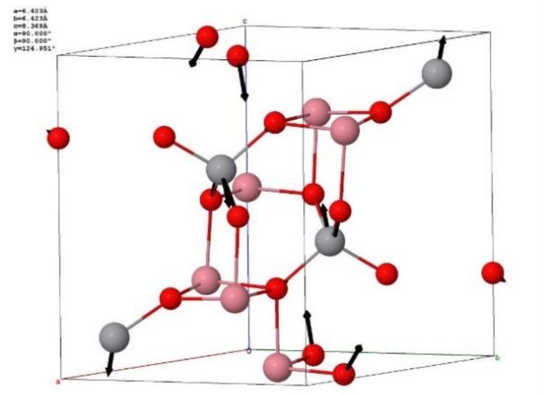 A_g^{10} (817 cm^{-1})	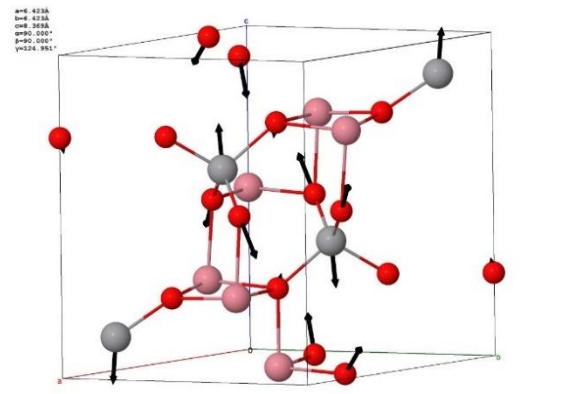 B_{3g}^{11} (839 cm^{-1})

* Vibration amplitudes vary with the particular cation, but the direction does not. In this table, Co is used.

Table S3. Atomic motions of the silent modes of $M_3V_2O_8$ (M = Ni, Co)*. The black vectors were calculated with VASP using the primitive unit-cell and then represented with Jmol. M is in pink, V in gray and O in red.

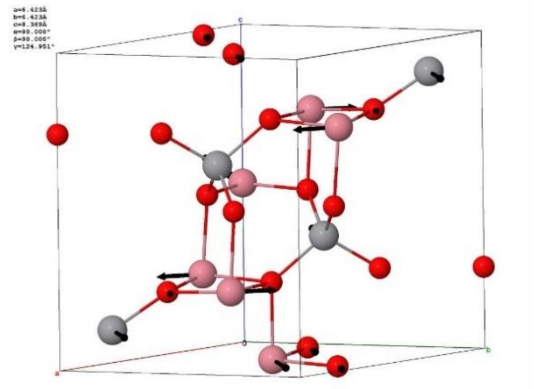 A_u^1 (133 cm^{-1})	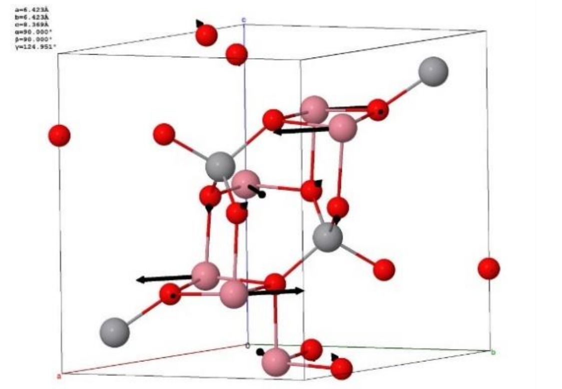 A_u^2 (153 cm^{-1})
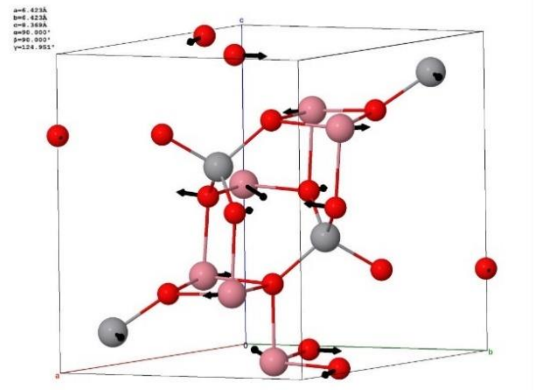 A_u^3 (246 cm^{-1})	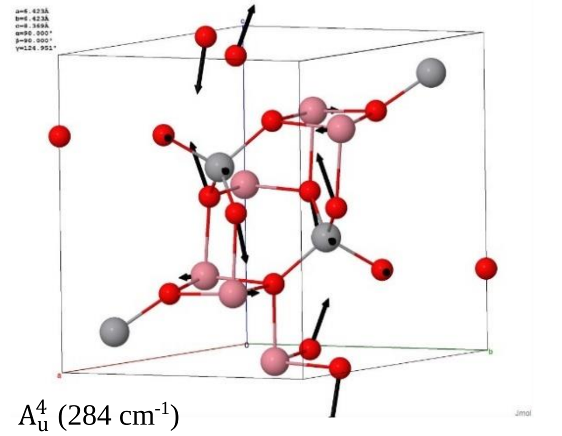 A_u^4 (284 cm^{-1})

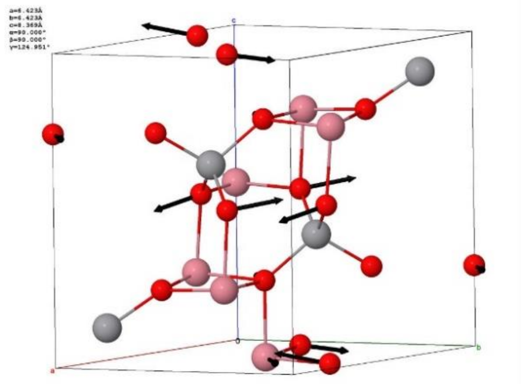 A_u^5 (347 cm^{-1})	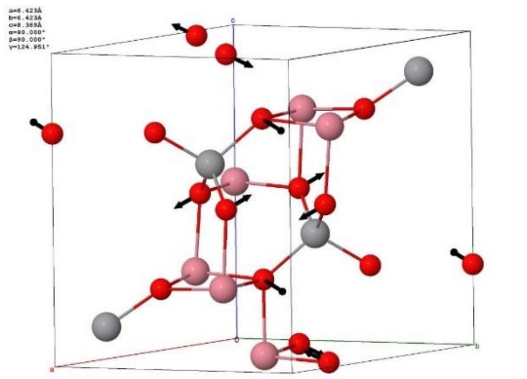 A_u^6 (407 cm^{-1})
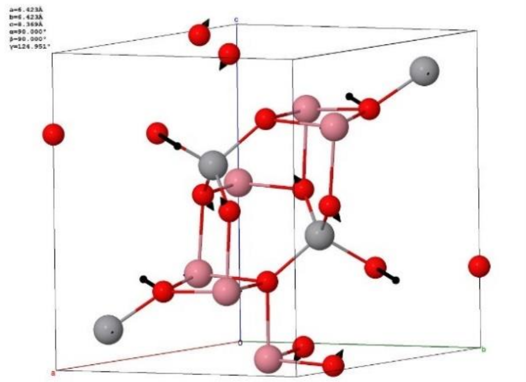 A_u^7 (461 cm^{-1})	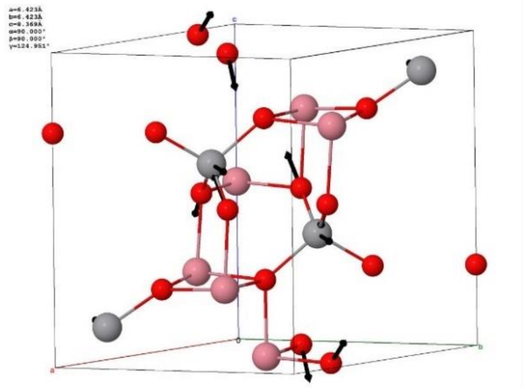 A_u^8 (823 cm^{-1})

* Vibration amplitudes vary with the particular cation, but the direction does not. In this table, Co is used.

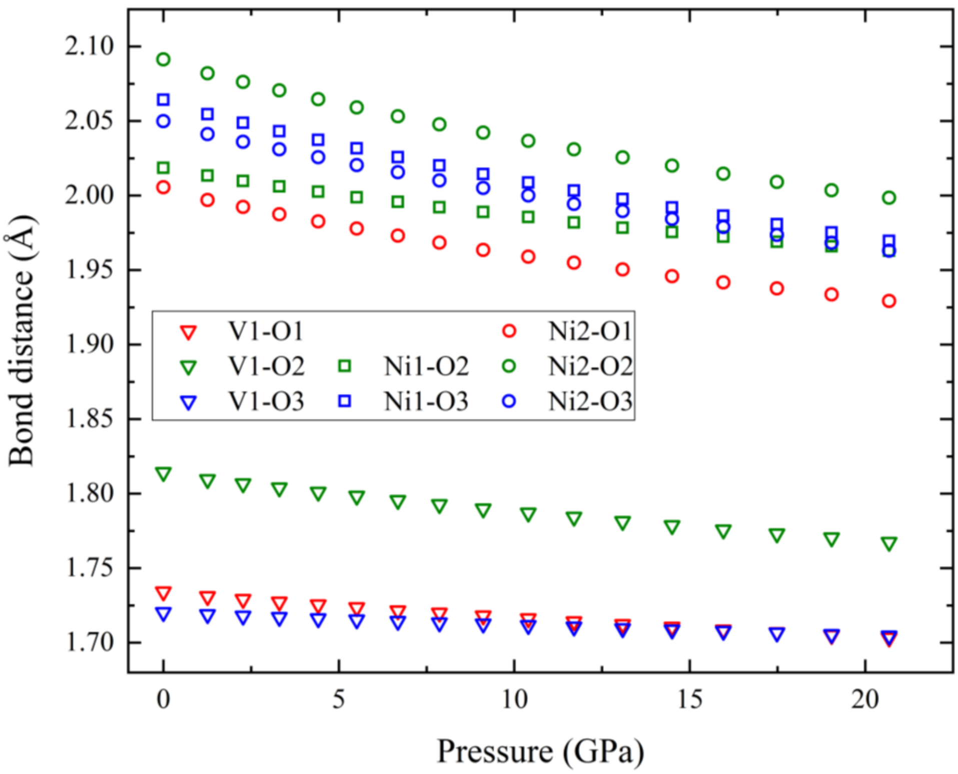

Figure S1. DFT calculated bond distances under HP for orthorhombic $Ni_3V_2O_8$ (space group *Cmca*).

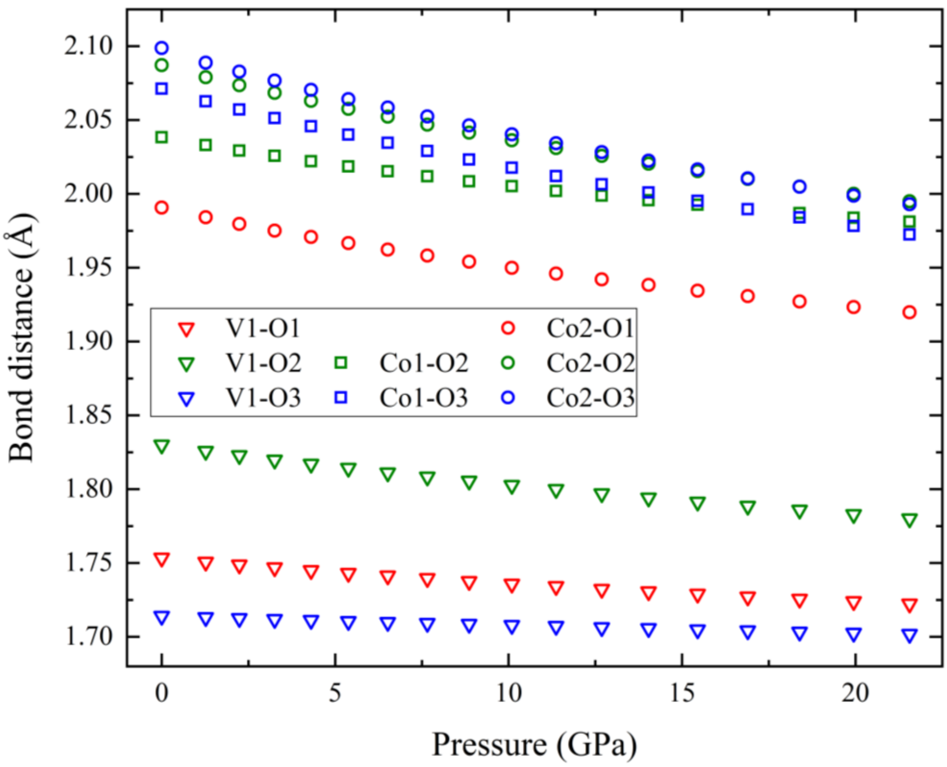

Figure S2. DFT calculated bond distances under HP for orthorhombic $Co_3V_2O_8$ (space group *Cmca*).
